# Estimating the contribution of transmission in primary healthcare clinics to community-wide TB disease incidence, and the impact of infection prevention and control interventions, in KwaZulu-Natal, South Africa

**DOI:** 10.1136/bmjgh-2021-007136

**Published:** 2022-04-08

**Authors:** Nicky McCreesh, Aaron S Karat, Indira Govender, Kathy Baisley, Karin Diaconu, Tom A Yates, Rein MGJ Houben, Karina Kielmann, Alison D Grant, Richard White

**Affiliations:** 1 TB Centre, London School of Hygiene and Tropical Medicine, London, UK; 2 The Institute for Global Health and Development, Queen Margaret University, Musselburgh, UK; 3 Africa Health Research Institute, School of Laboratory Medicine & Medical Sciences, College of Health Sciences, University of KwaZulu-Natal, Durban, KwaZulu-Natal, South Africa; 4 Department of Infectious Disease, Faculty of Medicine, Imperial College London, London, UK; 5 School of Public Health, University of the Witwatersrand, Johannesburg, South Africa

**Keywords:** tuberculosis, HIV, mathematical modelling, epidemiology, public health

## Abstract

**Background:**

There is a high risk of *Mycobacterium tuberculosis* (*Mtb*) transmission in healthcare facilities in high burden settings. WHO guidelines on tuberculosis (TB) infection prevention and control (IPC) recommend a range of measures to reduce transmission in healthcare settings. These were evaluated primarily based on evidence for their effects on transmission to healthcare workers in hospitals. To estimate the overall impact of IPC interventions, it is necessary to also consider their impact on community-wide TB incidence and mortality.

**Methods:**

We developed an individual-based model of *Mtb* transmission in households, primary healthcare (PHC) clinics, and all other congregate settings. The model was parameterised using data from a high HIV prevalence community in South Africa, including data on social contact by setting, by sex, age, and HIV/antiretroviral therapy status; and data on TB prevalence in clinic attendees and the general population. We estimated the proportion of disease in adults that resulted from transmission in PHC clinics, and the impact of a range of IPC interventions in clinics on community-wide TB.

**Results:**

We estimate that 7.6% (plausible range 3.9%–13.9%) of non-multidrug resistant and multidrug resistant TB in adults resulted directly from transmission in PHC clinics in the community in 2019. The proportion is higher in HIV-positive people, at 9.3% (4.8%–16.8%), compared with 5.3% (2.7%–10.1%) in HIV-negative people. We estimate that IPC interventions could reduce incident TB cases in the community in 2021–2030 by 3.4%–8.0%, and deaths by 3.0%–7.2%.

**Conclusions:**

A non-trivial proportion of TB results from transmission in clinics in the study community, particularly in HIV-positive people. Implementing IPC interventions could lead to moderate reductions in disease burden. We recommend that IPC measures in clinics should be implemented for their benefits to staff and patients, but also for their likely effects on TB incidence and mortality in the surrounding community.

**WHAT IS ALREADY KNOWN ON THIS TOPIC**

- Healthcare workers are at increased risk of tuberculosis (TB) in high burden settings, suggesting that there is a high rate of *Mycobacterium tuberculosis* transmission in healthcare facilities.
- A range of infection prevention and control (IPC) measures exist, but most evidence of their potential impact comes from studies of healthcare workers only, in hospital settings, with little known about the potential effects of IPC interventions on community-wide TB incidence.

**WHAT THIS STUDY ADDS**

- We estimate that in a high TB burden, high HIV prevalence community in KwaZulu-Natal, South Africa, 7.6% (plausible range 3.9%–13.9%) of TB in adults results directly from transmission in primary healthcare (PHC) clinics.
- IPC interventions in PHC clinics could reduce the number of incident TB cases in the community in 2021–2030 by 3.4%–8.0%, and the number of deaths by 3.0%–7.2%.

**HOW THIS STUDY MIGHT AFFECT RESEARCH, PRACTICE AND/OR POLICY**

- This study provides support for the implementation of IPC interventions in clinics, demonstrating that they could not only directly reduce TB risk in staff and clinic attendees, but could also lead to moderate reductions in overall TB incidence and mortality in the community.

## Introduction

Tuberculosis (TB) is a major global public health problem, killing an estimated 1.4 million people in 2019.^1^ There is a high risk of transmission in healthcare facilities in high TB burden settings, evidenced by the elevated rate of TB in healthcare workers.^2^ Updated WHO guidelines on TB infection prevention and control (IPC) recommend a wide range of measures to reduce transmission in healthcare and institutional settings, ranging from triaging people with TB symptoms to installing ultraviolet germicidal irradiation (UVGI) systems.^2^ These measures were evaluated and implemented as recommendations in the guidelines primarily based on evidence on their effects on risk to healthcare workers, and in hospitals settings.

Protecting healthcare workers should be a key concern of TB control programmes. However, the motivation for, and potential benefits of, IPC interventions in clinics extend beyond the reductions in disease burden among clinic staff. While healthcare workers and other clinic staff are at the highest risk of infection in clinics, due to their longer durations of exposure, the numbers of patients and other clinic attendees are far higher than numbers of staff. It is therefore likely that a large proportion of clinic-acquired TB is in patients and other clinic attendees. As a consequence, it is imperative that the impact on TB incidence in the wider community is considered when estimating the likely impacts of IPC measures.

Estimating the contribution of transmission in clinics (or other congregate settings) to overall community-wide disease burden is challenging. Taylor *et al* used data on ventilation rates and a Wells-Riley approach to estimate a 0.03% risk of infection to patients per clinic visit. This approach is heavily dependent on estimates of mean quanta production rates, however, about which there is considerable uncertainty (their sensitivity analysis gave a wide range of 0.02%–0.35%). Andrews *et al* also used a Wells-Riley based approach to determine infection risk by location (although not clinics), but removed the dependence on an assigned value for the quanta production rate by using data on contact time in multiple types of location, and calibrating their model to the prevalence of infection by age.^3^

In this work, we used a social contact data-based approach similar to that adopted by Andrews *et al*, but used an individual-based model (IBM) that includes HIV/antiretroviral therapy (ART) and TB disease development and resolution, and calibrated the model to overall disease incidence. This allowed us to determine the contribution of primary healthcare (PHC) clinics not only to the incidence of infection, but also to community-wide disease incidence and mortality. This is important for determining the true contribution of clinics-based transmission to disease burden, due to the increased rates of clinic attendance by people at increased risk of progression to disease.^4^ We also incorporated empirical data on the increased prevalence of TB in PHC clinic attendees compared with the general population, something that acts to amplify transmission in clinics.^4^ The study community used was the population living in the catchment area of two PHC clinics in KwaZulu-Natal province, South Africa.

The IPC interventions we simulated were identified and parameterised through a rigorous multidisciplinary approach. This work forms part of the *Umoya omuhle* project, that used a whole systems approach to study IPC in primary healthcare facilities in South Africa. As part of the project, system dynamics modelling was used to identify potential IPC interventions that local policy makers and health professionals active at clinic and province levels ranked highly in terms of both feasibility of implementation and perceived likely impact on overall and multidrug resistant (MDR) *Mycobacterium tuberculosis* (*Mtb*) transmission in clinics.^5^ The impact of the interventions on the rate of *Mtb* transmission to clinic attendees was then estimated using an IBM that simulated the flow of patients through clinics, and ventilation rates and infection risk in clinic waiting areas.^6^

## Methods

### Social contact data

#### Data collection

A social contact survey was conducted in the catchment areas of two primary healthcare clinics in the southern section of the Africa Health Research Institute (AHRI) demographic surveillance area (DSA),^7^ in March–December 2019. Three thousand and ninety-three adults (aged 18 years and over) were sampled, stratified by local area.

Respondents were asked to list all indoor locations visited and transport used on an assigned day in the week before the survey. For each location visited (including their own home) and transport used, they were asked for further details, including the type of location, the duration of time they spent there and the number of other people present. Respondents were also asked the number of times they had visited clinics in the 6 months before the interview, and how long they spent at the clinic and a cross-sectional estimate of the number of people present on their last visit.

Further details of the social contact survey are given in the online supplemental material and in McCreesh *et al*.^8^

10.1136/bmjgh-2021-007136.supp1 Supplementary data

#### Patient and public involvement

The social contact data collection was discussed with, and approved by, AHRI’s community advisory board prior to finalisation of the study protocol.

#### Analysis

For each location visited on the assigned day, adult contact times were calculated as the reported number of adults present (capped at a maximum of 100) multiplied by the duration of time spent at the location. Respondents who reported being HIV positive were considered to be HIV positive. Otherwise, respondents were considered to be HIV negative/unknown.

### Transmission model

We developed an IBM of social contact behaviour, *Mtb* transmission by location, TB disease development and treatment, and HIV and ART. The model population was designed to represent the social contacts survey study population. Simulated individuals were created at age 15 years, and died at age 80 years, with additional TB, HIV and background mortality occurring between those ages. People aged <15 years were not simulated, as the risk of transmission from children is low,^9^ and contact data were not available from children from the study population.

**Figure 1:**
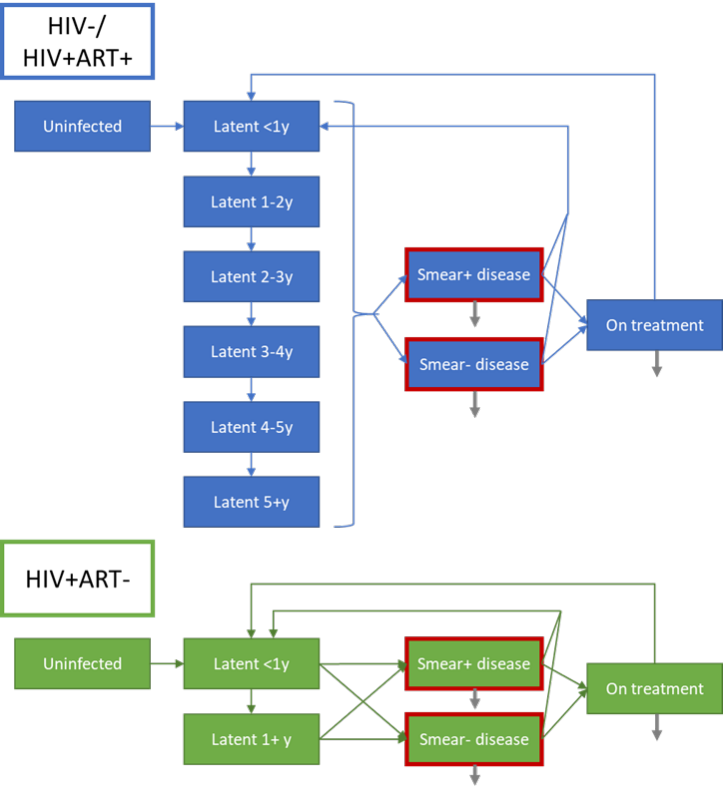
Simulated TB states. Blue and green boxes show the natural history for HIV−/HIV+ART+ and HIV+ART− individuals respectively. Grey arrows indicate tuberculosis mortality. Red outlines indicate infectious states. ART, antiretroviral therapy; TB, tuberculosis.

Individuals could be uninfected with *Mtb*, have a latent infection, have smear-negative disease, have smear-positive disease or be on treatment for TB (figure 1). Drug sensitivity was represented as non-MDR-TB or MDR-TB. HIV was also simulated, and individuals could be HIV-negative; HIV-positive, not on ART; or HIV-positive, on ART.

Each simulated individual was a member of a household, with the household size distribution taken from empirical data.^7^ Each individual had the same amount of contact time with each household member each month. Mean clinic contact time per month in the model varied by sex and HIV/ART status strata, and within those strata, by whether someone was assigned to a high or low clinic visiting group. Empirical data from TB prevalence surveys conducted among attendees at the two clinics and in the general population in the DSA found a 86% (95% CI 10% to 310%) higher prevalence of TB among the clinic attendees.^10^ Mean clinic contact time in the model could therefore also be higher in people with untreated TB disease, to allow the model to be fitted to the prevalence of TB in clinic attendees relative to the general population. Finally, contact time occurring in all other indoor locations (including transport) was simulated, varying by sex, age group and HIV/ART status. Contact time was parameterised using data from the social contact survey. Table 1 shows the simulated parameter values for mean monthly contact hours occurring between household members, in clinics, and in other congregate settings, by simulated population group, for people without TB.

**Table 1:** Simulated parameter values for mean monthly contact hours occurring between household members, in clinics, and in other congregate settings, by simulated population group, for people without TB

Sex	Age group (years)	HIV/ART status	Estimated mean contact hours per month		
			Household members	Clinics	Other congregate settings
Male	15–29	HIV−/HIV+ not on ART	3175	50	2315
		HIV+ on ART	3175	99	1939
	30–49	HIV−/HIV+ not on ART	3175	50	1636
		HIV+ on ART	3175	99	1260
	50–79	HIV−/HIV+ not on ART	3175	50	1567
		HIV+ on ART	3175	99	1191
Female	15–29	HIV−/HIV+ not on ART	3175	91	2394
		HIV+ on ART	3175	138	2017
	30–49	HIV−/HIV+ not on ART	3175	91	1714
		HIV+ on ART	3175	138	1338
	50–79	HIV−/HIV+ not on ART	3175	91	1646
		HIV+ on ART	3175	138	1269

ART, antiretroviral therapy; TB, tuberculosis.

A baseline rate of transmission per minute contact between each person with untreated TB and each person uninfected or latent person was simulated. This was adjusted according to the smear status of the person with TB; whether the person at risk was uninfected or had a latent infection (giving partial protection against reinfection), and, if latent, by their HIV/ART status; and the assumed mean rate of ventilation in locations of that type (household, clinic or other).

The model was fitted by hand (by manually varying the values of input parameters) to empirical data from the social contact study population and from KwaZulu-Natal province, generating a single hand fit. Fitting targets included the age and sex distribution of the population; HIV prevalence and ART coverage by age and sex; and TB incidence, mortality and treatment coverage. Details of the uncertainty analyses are given in the section ‘Uncertainty analyses’.

A full description of the model and parameters is given in the online supplemental material.

#### Interventions

Seven potential IPC interventions had been identified in qualitative research and system dynamics modelling exercises conducted as part of the *Umoya omuhle* project.^5^ The effect of the interventions on patient contacts and infection risk in eight clinics were estimated in previous modelling work, using a within-clinics model that simulated the flow of patients through clinics, and ventilation rates and infection risk in clinic waiting areas.^6^ Results were aggregated across all clinics, giving a single estimate for the effect of each intervention. The interventions were:

1. *Windows and doors*. Ensuring windows and doors in waiting areas are kept open at all times. This was estimated to reduce the rate of transmission to clinic attendees by 55% (IQR 25%–72%).
2. *Retrofits*. Building retrofits are changes to the building to improve ventilation rates. This could include installing lattice brickwork or whirlybird fans. Due to the large amount of variation between clinic spaces in the types of building retrofits that would be suitable, and the lack of sufficient data on the effects of the retrofits on ventilation rates in different types of spaces, we did not model specific retrofits or packages of retrofits. Instead, in the within clinics model, we simulated an undefined package of retrofits that are sufficient to increase air changes per hour to a minimum of 12 in all waiting rooms, chosen in line with WHO guidelines.^2 11^ This was estimated to reduce the rate of transmission to clinic attendees by 45% (IQR 16%–64%).
3. *UVGI system*. We assumed in this intervention that appropriate and well maintained UVGI systems are installed in all indoor clinic waiting areas. This was estimated to reduce the rate of transmission to clinic attendees by 77% (IQR 64%–85%).
4. *Masks*. We simulated a scenario where 70% of patients wear surgical masks 90% of the time. This was estimated to reduce the rate of transmission to clinic attendees by 47% (IQR 42%–50%).
5. *CCMDD coverage*. South Africa’s Central Chronic Medicine Dispensing and Distribution (CCMDD) programme is designed to allow patients with stable chronic health conditions to collect their medicines from convenient locations, such as local pharmacies.^12^ This means that they do not need to queue at clinics unnecessarily. The purpose of this intervention was to increase the utilisation of CCMDD and similar programmes by eligible patients, and to ensure that pick-up points do not require patients to queue at clinics. This was estimated to reduce mean clinic contact time per visit by 28% (IQR 9%–42%) for patients on ART and 13% (8%–19%) for all other patients, reducing the overall rate of transmission to clinic attendees by 22% (IQR 12%–32%).
6. *Queue management system with outdoor waiting area*. This intervention combined a large, well ventilated, covered outdoor waiting area with a queue management system. This was estimated to reduce the rate of transmission to clinic attendees by 83% (IQR 76%–88%).
7. *Appointment system*. In this intervention, we simulated a date–time appointment system to reduce clinic overcrowding, through spacing out the arrival times of patients. This was estimated to reduce the overall rate of transmission to clinic attendees by 62% (IQR 45%–75%).

The estimated effects of the interventions on patient contacts and infection risk in clinics from the within-clinics model were used to parameterise the effects of the interventions on contact rates and transmission probabilities in clinics in this model, allowing their wider effects on community-level disease incidence to be estimated. The interventions were implemented in the model from 2021. Full details are given in the online supplemental material.

#### Uncertainty analyses

A number of univariate sensitivity analyses were conducted, exploring the effects of uncertainty in clinic contact time, the proportion of disease from transmission between household members, ventilation rates in clinics, the prevalence of TB in clinic attendees relative to the general population, the rate at which people switch between the high and low clinic visiting groups, clinic visiting rates in HIV-people who are not on ART and future HIV incidence. These sensitivity analyses were used to construct a plausible range around the estimated proportion of disease that results from transmission in clinics, and estimated intervention impact. Full details are given in the online supplemental material.

### Proportion of disease from transmission in clinics that is in clinic staff

In the mathematical model, we only consider transmission to adult clinic attendees. Clinic staff are also at risk of infection in clinics, however. We used a simple method to obtain a rough estimate of the proportion of disease that results from transmission in clinics that is in clinic staff, assuming that all clinic staff who are at elevated risk of infection from transmission in clinics have the same exposure to TB outside the clinic as the general population, and that all excess TB in clinic staff results from transmission in clinics. Full details are given in the online supplemental material.

## Results

### Social contact data

A total of 1704 individuals were interviewed. A description of respondent characteristics and reported contact time is given in the online supplemental material.

### Fit to data

**Figure 2:**
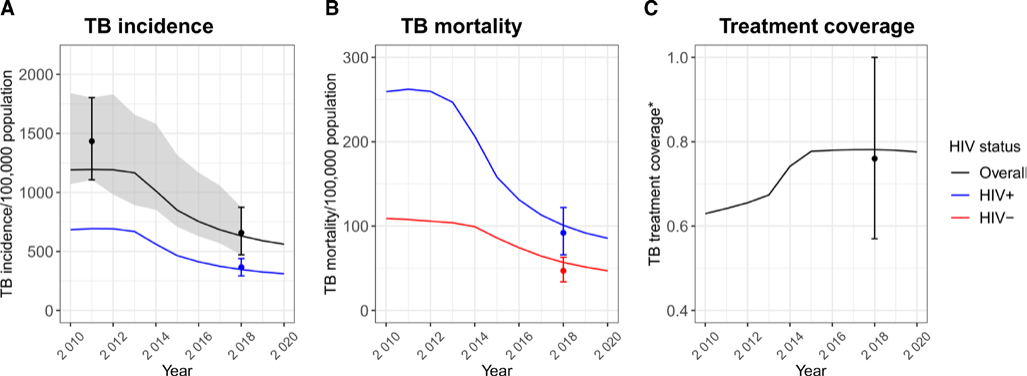
Model fit to estimated (A) TB incidence, (B) mortality and (C) treatment coverage. *Treatment coverage is calculated as the ratio of the number of people starting treatment in a year to the estimated number of people developing TB in the same year. Lines over time show model output. Points and error bars show the fitting targets, based on empirical data. The ribbon in plot (A) shows the empirical estimates over time. Empirical estimates over time were not available for KwaZulu-Natal for the other fitting outputs shown here. TB, tuberculosis.

The model fit well to all the fitting targets, in the main scenario and the sensitivity analyses scenarios (figure 2 and online supplemental tables S1 and S2).

### Proportion of disease from transmission in clinics

**Figure 3:**
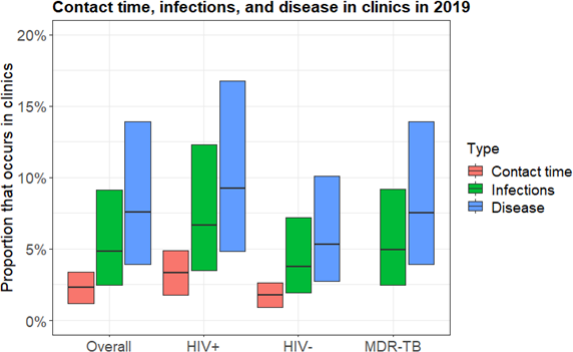
The estimated proportion of contact time and infections that occurs in clinics, and the proportion of disease that resulted from transmission in clinics in the study population in 2019, overall, in HIV-positive people, HIV-negative people and for MDR-TB. For contact time, the central bar shows the proportion for the best estimate scenario, and the range of the bars shows the proportions in the contact time in clinics sensitivity analysis. For infections and disease, the central horizontal bar shows the best estimate, and the range of the bars shows the most extreme results from the sensitivity analyses. MDR, multidrug resistant; TB, tuberculosis.

Overall, 2.3% (plausible range 1.2%–3.4%) of contact time by adults in the model occurred in clinics 2019, leading to 4.9% (2.5%–9.1%) of overall and MDR infections, and 7.6% (3.9%–13.9%) of overall and MDR disease (figure 3). The proportion of all TB disease that resulted from transmission in clinics was higher in HIV-positive people, at 9.3% (range 4.8%–16.8%), and lower in HIV-negative people, at 5.3% (2.7%–10.1%).

### Intervention impact

**Figure 4:**
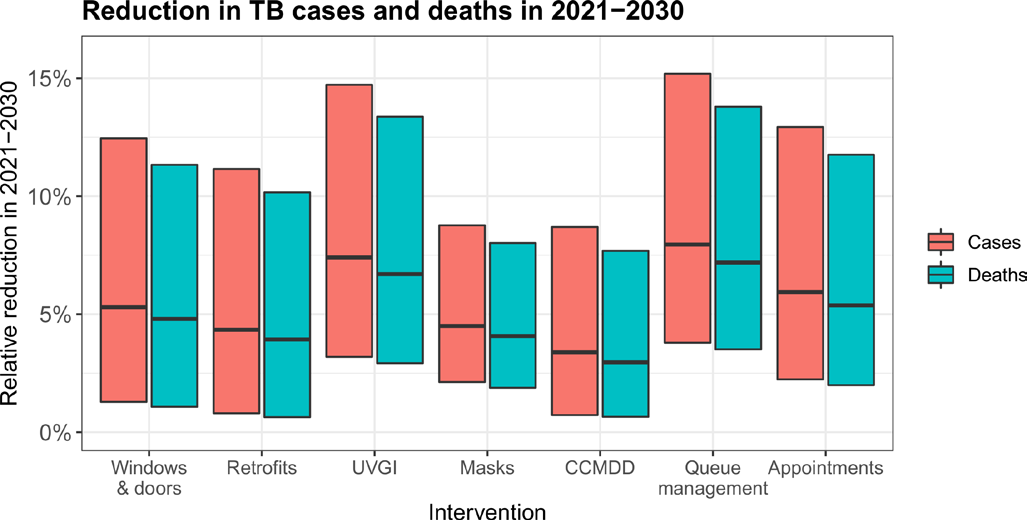
The estimated reduction in TB cases and deaths in the study population in 2021–2030 resulting from the proposed infection control interventions. The central horizontal bar shows the best estimate, and the range of the bars shows the most extreme results from the sensitivity analyses. CCMDD, Central Chronic Medicine Dispensing and Distribution; TB, tuberculosis; UVGI, ultraviolet germicidal irradiation.

Opening windows and doors reduced the total number of incident TB cases in the community in 2021–2030 by 5.3% (range 1.3%–12.5%), simple clinic retrofits by 4.3% (0.8%–11.2%), UVGI systems by 7.4% (3.2%–14.7%), surgical mask wearing by patients by 4.5% (2.1%–8.8%), increased CCMDD coverage by 3.4% (0.7%–8.7%), queue management systems with outdoor waiting areas by 8.0% (3.8%–15.2%) and appointment systems by 5.9% (2.2%–12.9%) (figure 4). Reductions in MDR-TB cases were similar to reductions in all TB cases (online supplemental material, figure S6).

Reductions in TB deaths were 9.5%–12.6% lower than reductions in cases, reflecting the time lag between developing disease and dying from TB. The reductions in deaths ranged from 3.0% (range 0.7%–10.1%) for increased CCMDD coverage, to 7.2% (range 2.7%–13.8%) for queue management systems with outdoor waiting areas.

### Proportion of disease from transmission in clinics that is in clinic staff

We estimate that in the study community, an average of 7.1% (95% plausible range 2.3%–16.7%) of all disease in adults resulting from transmission in clinics occurs in clinic staff.

## Discussion

In this paper, we estimate that 7.6% (plausible range 3.9%–13.9%) of TB in adults results directly from transmission in PHC clinics in a high HIV prevalence, rural/peri-urban setting in KwaZulu-Natal, South Africa. The proportion is higher in HIV-positive people, at an estimated 9.3% (range 4.8%–16.8%), compared with 5.3% (2.7%–10.1%) in HIV-negative people. We estimate that IPC interventions in PHC clinics could reduce the number of incident TB cases in the community in 2021–2030 by 3.4%–8.0%, and deaths by 3.0%–7.2%. These findings further strengthen the case for an increased emphasis on IPC in clinics, not just as a tool for protecting clinic staff and patients, but as method for reducing community-wide TB incidence and mortality.

Our findings highlight the importance of considering contact saturation^13^ and the population at risk when estimating the proportion of disease that results from transmission in different types of setting. We estimate that 4.9% of infections occur in clinics, more than double the estimated 2.3% of contact time occurring in clinics. This reflects the fact that contacts between household members are repeated, reducing their overall importance to transmission, and increasing the importance of other settings. It also reflects higher rates of clinic attendance by people with potentially infectious TB. We estimate that an even higher proportion of disease results from transmission in PHC clinics, at 7.7%. This reflects both the additional effects of contact saturation and repeated infections between household members, but also the higher rates of clinic attendance by HIV-positive people, who are at increased risk of progression to disease following infection.

We parameterised our model to data from a high HIV prevalence, high TB burden, rural/peri-urban setting in KwaZulu-Natal, South Africa. The proportion of TB that results from transmission in clinics, and the impact of IPC interventions on community-wide TB incidence, is likely to vary by setting, depending on a range of factors. These include: the proportion of people’s contact time that occurs in clinics; the prevalence of HIV and other TB risk factors, and how clinic visiting and other contact behaviour varies between people with different risk factor profiles; and the number of clinic visits people with TB need to make before receiving a diagnosis. Social contact data from sub-Saharan Africa are limited,^14^ however, our estimate of the proportion of social contact that occurs in clinics falls with the range found by other studies.^15 16^

The social contact data used in our model were collected in 2019, before the start of the COVID-19 pandemic. Comparable social contact data were collected from the same study community in June–August 2020, during the pandemic, and suggested that reductions in contact time in clinics may have been smaller than reductions in other congregate locations,^8^ possibly increasing the importance of transmission in clinics to overall disease burden during this period. The IPC interventions we simulated were also designed and parameterised before the start of the pandemic, and changing views on IPC may have changed the relative impact of the different interventions over longer time periods. For instance, the acceptability to patients of mask wearing may increase, increasing the coverage that can be achieved.

The main sources of uncertainty in our estimates come from three key inputs into the model: the proportion of contact time that occurs in clinics, the prevalence of TB in clinic attendees relative to the general population and ventilation levels in clinics relative to other congregate settings. Additional data collection in those three areas would be valuable, both in allowing us to reduce the uncertainty in our estimates, and in allowing similar estimates to be made for other settings.

There are a number of limitations to our work. First, we do not explicitly consider infection to or from clinic staff. This may have led to us underestimating the proportion of disease that results from transmission in clinics, due to amplification of transmission in clinics by clinic staff, and to us slightly underestimating the impact of the interventions on community-wide TB incidence. The underestimates are likely to have been small however, as we estimate that only 7.1% (95% plausible range 2.3%–16.7%) of all disease that results from transmission in clinics is in clinic staff, and contact time between clinic attendees and staff in clinics is much lower than contact time between clinic attendees. We also do not simulate children, as social contact data from children were not available. This will have had little effect on our estimates for adults, as the risk of *Mtb* transmission from children is low,^17^ but means that we cannot estimate the proportion of disease in children that comes from transmission in clinics.

We do not consider the effects of risk factors other than HIV, such as diabetes. People with diabetes and some other risk factors are both likely to visit clinics more frequently, and are at increased risk of progression to disease following infection. By not including these risk factors, we may have underestimated the proportion of disease that results from transmission in clinics.

Finally, the representation of MDR in the model is relatively simple. We implicitly assume that with high coverage of Xpert MTB/RIF,^18^ drug resistance is diagnosed for the majority of people at the same clinic visit as their TB is diagnosed. We therefore assumed that people with infectious MDR-TB spend no more time in clinics than people with infectious non-MDR-TB, and so the proportion of TB from transmission in clinics does not vary by drug resistance status. We were not able to explore the effects of this assumption, due to the very low incidence of MDR-TB, and the need to use an IBM to accurately capture patterns of social contact behaviour. Future work should investigate if and if so, how, the proportion of MDR-TB that results from transmission in clinics varies from the proportion of non-MDR-TB.

To conclude, we estimate that in the setting studied, 7.7% (4.0%–14.2%) of TB in adults is acquired through transmission in PHC clinics, and that IPC interventions in clinics could reduce the total number of incident TB cases in the community in 2021–2030 by 3.4%–8.0%. Given the relative ease of implementing IPC measures in clinics, compared with many other proposed TB control measures, we suggest that IPC measures in clinics should be considered to be ‘low hanging fruit’, and should be implemented both for their benefits to staff, but also for their likely effects on wider TB incidence and mortality.

## Data Availability

Data are available upon reasonable request. Data are available in a public, open access repository. Data are available upon reasonable request. The mathematical model used in this work is available from https://github.com/NickyMcC/ClinicTransmission. Model The social contact data used in this work will be made available from https://datacompass.lshtm.ac.uk/.
