# Supplementary material for: Estimating the contribution of transmission in primary healthcare clinics to community-wide TB disease incidence, and the impact of infection prevention and control interventions, in KwaZulu-Natal, South Africa
Source: BMJ Glob Health. 2022 Apr 8;7(4):e007136. doi: 10.1136/bmjgh-2021-007136 (PMC8995945; doi:10.1136/bmjgh-2021-007136)
Supplement: Supplementary data [file bmjgh-2021-007136supp001.pdf]

Supplemental material: Estimating the contribution of primary healthcare clinics to community-wide TB disease incidence, and the impact of infection prevention and control interventions, in KwaZulu-Natal, South Africa

Contents

1 Social contact data 4
1.1 Methods 4
1.1.1 Data collection 4
1.1.2 Analysis 5
1.2 Results 6
1.2.1 Recruitment 6
1.2.2 Time spent in own home 7
1.2.3 Clinic visiting and contact time 7
1.2.4 Contact in other locations 9
2 Model description 9
2.1 Key 9
2.2 Agents 9
2.2.1 People 9
2.2.2 Households 10
2.3 Model initialisation 12
2.4 Model scheduling 12
2.5 Model runs and calibration 12
2.6 Demography 13
2.6.1 Fitting targets 13

2.7 Social contact ... 13
2.7.1 Household members ... 14
2.7.2 Clinics ... 14
2.7.3 Other locations ... 14
2.7.4 Fitting targets ... 14
2.8 Ventilation ... 15
2.9 Tuberculosis ... 15
2.9.1 Disease states ... 15
2.9.2 Drug resistance ... 15
2.9.3 Disease progression ... 16
2.9.4 Treatment ... 16
2.9.5 Mortality ... 17
2.9.6 Prevalence of infection in 15-year olds ... 17
2.9.7 Changes in TB natural history parameters over time ... 18
2.9.8 Fitting targets ... 18
2.10 *Mtb* transmission ... 18
2.10.1 Individual-level variation in infectiousness ... 19
2.10.2 Household members ... 19
2.10.3 Clinics ... 20
2.10.4 Other locations ... 21
2.11 HIV/ART ... 22
2.11.1 Effects on TB ... 23
2.11.2 Changes in HIV parameters over time ... 23
2.11.3 Fitting targets ... 26
2.12 Interventions ... 26
2.13 Results calculations ... 29
2.14 Uncertainty analyses ... 29
2.15 Input parameters ... 32

2.16 Model fitting targets ... 48

3 Model results ... 52

3.1 Calibrated input parameter values ... 52

3.2 Fit to data ... 54

3.3 Results by uncertainty analysis scenario ... 58

3.3.1 Proportion of disease from transmission in clinics ... 58

3.3.2 Intervention impact ... 58

4 Proportion of disease from transmission in clinics that is in clinic staff ... 61

4.1 Methods ... 61

4.2 Results ... 62

5 References ... 62

6 Acknowledgements ... 65

1 Social contact data

1.1 Methods

1.1.1 Data collection

A social contact survey was conducted in the catchment areas of two primary health clinics in the southern section of the Africa Health Research Institute (AHRI) demographic surveillance area (DSA), between 28th March 2019 and 9th December 2019. 3090 adults (aged 18 and over) were sampled, stratified by local area.

Respondents were asked if they knew their HIV status. Respondents who reported being HIV-positive were asked if they were on anti-retroviral therapy (ART).

Respondent household size was extracted from existing DSA data.

Respondents were asked to list all indoor locations visited and transport used on an assigned day in the week before the survey. For each location visited (including their own home), they were asked for further details, including:

- What type of location it was (options included 'own home' and 'clinic')
- How long they spent there
- How many people (adults and children) were there, halfway through the time they were there
- How many of those people were children aged <15 years

For each use of transport reported, they were asked for further details, including:

- What type of transport it was
- How long the journey took
- How many people (adults and children) were on the vehicle at the start of the trip
- How many of those people were children aged <15 years

Respondents were also asked for additional details on their clinic visiting behaviour during the six months prior to the interview, including:

- The number of days on which they had visited a clinic for their own health in the past six months
- The number of days on which they had visited a clinic for on the behalf of someone else (e.g. to collect a prescription) in the past six months, not included any visits that were also made for their own health

- The number of days on which they had accompanied someone else to a clinic in the past six months, not including any visits that were also made for their own health and/or on behalf of someone else

Finally, respondents were asked when their last visit to a clinic was, and, if it was within the past two years, they were asked for the following information about their last visit:

- How long they spent at the clinic
- How many people (adults and children) were there, halfway through the time they were there
- How many of those people were children aged < 15

Further details of the social contact survey are given in McCreesh *et al*[1].

1.1.2 Analysis

For each location visited on the assigned day, adult contact times were calculated as follows. Firstly, the number of adults present was calculated as the reported total number of people present, minus the reported number of children present. If this gave a value less than zero, it was set to missing. The number of adults present was then capped at 100, as above this value, it is unlikely that the respondent had sufficient contact with each adult present to allow transmission. The capped number of adults present was then multiplied by the duration of time that the respondent reported spending in the location, to give the adult contact time.

Estimates generated using the data on the respondent's last clinic visit were weighted by the reported number of clinic visits in the past six months.

Respondents who reported being HIV-positive were considered to be HIV-positive. Otherwise, respondents were considered to be HIV-negative/unknown.

1.2 Results

1.2.1 Recruitment

Of the 3090 people sampled for UO, 1723 (56%) were successfully contacted, 298 (10%) were dead or reported to have out-migrated, 1071 (35%) could not be contacted. Of those successfully contacted, 1704 (99%) completed an interview (Table S1).

		Sampled (%)	Contacted (%)	Dead or missing (%)	Responded (%)
**Sex**	Male	1582 (51%)	768 (45%)	175 (59%)	751 (44%)
	Female	1508 (49%)	955 (55%)	123 (41%)	953 (56%)
**Age group**	18-29	1163 (38%)	615 (36%)	132 (44%)	613 (36%)
	30-49	1117 (36%)	546 (32%)	105 (35%)	535 (31%)
	50+	810 (26%)	562 (33%)	61 (20%)	556 (33%)
**HIV status**	HIV negative or unknown				1210 (71%)
	HIV positive, not on ART				13 (1%)
	HIV positive, on ART				481 (28%)
**Household size**	1-3				293 (17%)
	4-6				426 (25%)
	7-9				429 (25%)
	10+				556 (33%)
**Total**		3090	1723	298	1704

Table S1. Social contacts survey respondent characteristics

1.2.2 Time spent in own home

Respondents reported spending a mean of 18.8 (95% CI 18.5-19.1) hours per day in their own home. This varied little by sex, age group, HIV status, or household size (Table S2).

		Mean hours spent in own home per day (95% CI)
**Sex**	Male	18.2 (17.8-18.7)
	Female	19.2 (18.9-19.6)
**Age**	18-29	18.1 (17.6-18.5)
	30-49	18.3 (17.8-18.9)
	50+	20.1 (19.6-20.5)
**HIV status**	Positive	18.7 (18.4-19.0)
	Negative/Unknown	19.1 (18.5-19.6)
**Household size**	1-3	18.4 (17.7-19.1)
	4-6	18.9 (18.3-19.4)
	7-9	19.0 (18.4-19.5)
	10+	18.8 (18.3-19.3)
**Overall**		18.8 (18.5-19.1)

Table S2. Mean reported time spent in own home, by sex, age, HIV status, and household size

1.2.3 Clinic visiting and contact time

1.2.3.1 Frequency of clinic visiting

Table S3 shows the estimated mean annual number of visits made to clinics, by sex, age, and HIV status, estimated from data on reported clinic visits in the past day, and in the past six months. Overall, there is little difference between the estimates calculated using the data collected using the two different recall durations. The exception to this is the estimates by sex, where there is a large difference in mean annual clinic visits by sex using the six-month recall data, but not the one-day recall data. However, the confidence intervals for the one-day recall estimates contain the estimated values for the six-month recall.

As there is no evidence that recall bias has had a large effect on the estimates, the six-month recall data are used to parameterise clinic visiting rates in the model, due to their greater precision.

McCreesh N, *et al. BMJ Global Health* 2022; 7:e007136. doi: 10.1136/bmjgh-2021-007136

		Mean annual clinic visits (95% CI)	
		One-day recall	Six-month recall
**Sex**	Male	7.8 (4.0-11.6)	5.1 (4.7-5.4)
	Female	7.7 (4.3-11.0)	9.3 (8.8-9.7)
**Age**	18-29	8.3 (4.0-12.7)	6.7 (6.1-7.3)
	30-49	8.9 (4.1-13.7)	7.9 (7.4-8.5)
	50+	5.9 (2.1-9.8)	7.7 (7.2-8.2)
**HIV status**	Negative/Unknown	6.7 (3.9-9.4)	6.0 (5.7-6.4)
	Positive	10.4 (5.0-15.7)	10.8 (10.2-11.4)
**Overall**		7.7 (5.2-10.2)	7.4 (7.1-7.7)

Table S3. Mean numbers of reported annual clinic visits by sex, age, and HIV status

1.2.3.2 Contact time

Table S4 shows the mean adult contact hours per clinic visit, by sex, age, and HIV status, estimated from data on reported clinic visits in the past day, and in the past six months. Overall, there is little difference between the estimates calculated using the data collected using the two different recall durations. It is plausible, however, that the accuracy of recall for time spent in the clinic and numbers of people present falls fairly rapidly over time, and therefore the one-day recall estimates are used for estimating adult contact hours for input into the model.

		Mean adult contact hours per visit (95% CI)	
		One-day recall	Last clinic visit*
**Sex**	Male	150 (71-230)	134 (121-147)
	Female	131 (65-197)	178 (165-190)
**Age**	18-29	116 (47-185)	163 (145-182)
	30-49	179 (84-275)	167 (149-185)
	50+	112 (15-209)	162 (149-175)
**HIV status**	Negative/Unknown	138 (76-201)	151 (138-164)
	Positive	143 (55-232)	182 (167-197)
**Overall**		140 (89-191)	164 (155-174)

Table S4. Mean reported adult contact hours per clinic visit, by sex, age, and HIV status. *Weighted by number of clinic visits in the past six months

1.2.4 Contact in other locations

Other locations are defined as indoor locations other than clinics and the respondents' own homes, and transport.

Table S5 shows the mean adult contact hours in other locations, by sex, age, and HIV status.

		Mean contact hours per day (95% CI)
**Sex**	Male	60 (51-70)
	Female	58 (48-68)
**Age**	18-29	75 (63-88)
	30-49	49 (38-61)
	50+	50 (37-62)
**HIV status**	Negative/Unknown	64 (56-72)
	Positive	47 (34-59)
**Overall**		59 (52-66)

Table S5. Mean reported contact hours per day in 'other' locations by sex, age, and HIV status

2 Model description

2.1 Key

Model parameter names are written in italics, with colour indicating whether the parameter is an input parameter, a parameter with a global model-wide value, calculated from input parameter(s) or other values, or an individual-level parameter, which can take a different value for each simulated person or household.

2.2 Agents

Two types of agents were simulated in the model, people and households.

2.2.1 People

The main state variables assigned to people in the model were:

- Unique ID – *person_ID*
- Age group – *age_group* (15-29, 30-49, 50-79)
- Sex – *sex* (male, female)
- Clinic visiting group – *clinic_group* (high, low)

- TB status – *TB_status* (uninfected, latent, smear+ disease, smear- disease, on treatment)
- TB strain – *TB_strain* (uninfected, non-multidrug resistant (non-MDR-TB), multidrug resistant (MDR-TB)
- Individual-level TB infectiousness – *infectiousness* (numeric, see section 'Individual-level variation in infectiousness)
- Location where last *Mtb* infection occurred – *infect_location* (uninfected, infected before creation, household, clinic, other location)
- HIV status – *HIV_status* (HIV-, HIV+ART-, HIV+ART+)

Other state variables were used to track individuals' histories in the model, for the purpose of creating model output.

2.2.2 Households

Households were simulated as agents, for the purpose of grouping people into households with the desired size distributions. Households had the following state variables:

- Unique ID – *hh_ID*
- Desired household size – *desired_hh_size*
- Current household occupancy – *current_hh_size*

Other temporary household-level state variables were used to store information on the disease states of household members when estimating transmission probabilities in the household (see section '*Mtb* transmission – Household members')

2.2.2.1 Household sizes

Empirical data were available from the study population on the number of people aged 15+ years in each household. An exponential distribution was fitted to data on the cumulative proportion of households below each size, and the distribution was sampled from and rounded up to the nearest whole number to create desired household sizes in the model (Figure S1). Mean household sizes were similar between the model and the empirical data both from the perspective of households (model=3.64, data=3.97), and from the perspective of individuals (model=6.75, data=6.55).

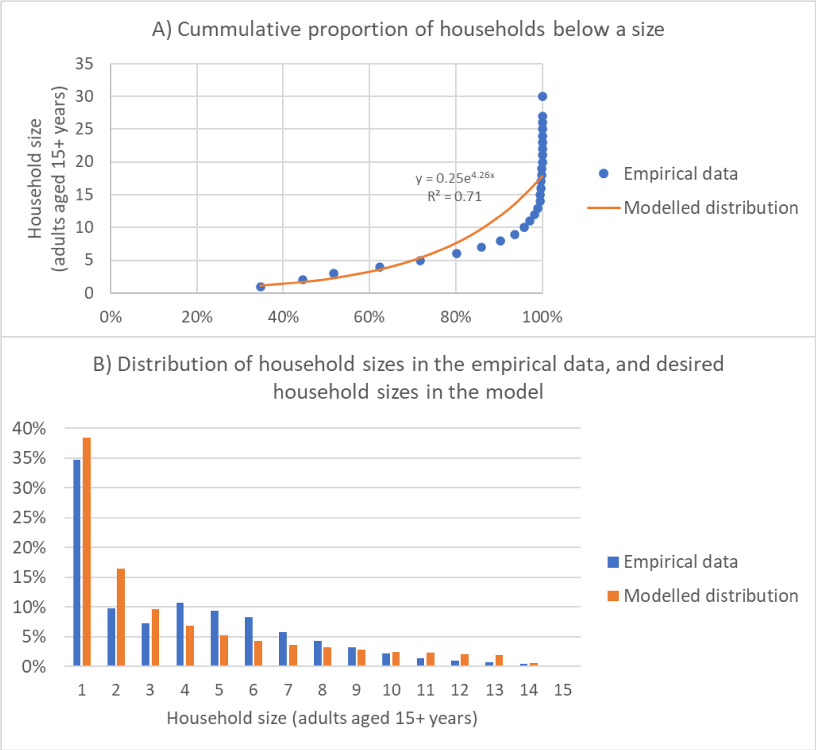

Figure S1. Distribution of household sizes in the empirical data, and desired household sizes in the model.

2.2.2.2 Household occupancy tracking and formation

To initialise the model, N empty households were created, where N = round (10,000 /*mean_hh_size*). Each empty household sampled a desired household size, *desired_hh_size*, from the exponential distribution (Figure S1), rounding up to the nearest whole number, and then created that number of people to populate the household, setting *current_hh_size* = *desired_hh_size*.

When people died, the household they were a member of reduced the value of *current_hh_size* by one. The household also added its *hh_ID* to the end of a list tracking households that are not at full occupancy.

When new people were created in the model, they checked the length of the list. If it was greater than one, the person joined the first household on the list. The household removed its *hh_ID* from the start of the list, and increased the value of *current_hh_size* by one.

If the length of the list was zero (i.e. there were no households that were not at full occupancy), then a new household was created, and the person joined it. The new household sampled a

desired_hh_size, and if *desired_hh_size* > *1*, it added itself to the tracking list (*desired_hh_size* – 1) times.

2.3 Model initialisation

To initialise the model, N empty households were created, where N = round (10,000 /*mean_hh_size*). Each empty household sampled a household size from an exponential distribution, rounding up to the nearest whole number (see section 'Household sizes' for details), and then created that number of people to populate the household. This gave an initial population size of approximately 10,000.

The newly created people were each assigned a *sex*, with a probability of 0.5 of being male and 0.5 of being female, and a *clinic_group* with and a probability of 0.5 of being 'high' and 0.5 of being 'low'. They were then assigned an *age_group*, with probabilities assigned by input parameters, and varying by sex; and an age, drawn from a uniform distribution between the minimum and maximum ages in their *age_group*. A random *infection_seed_proportion* were seeded with latent infection, with no risk of progression without reinfection. A random *tb_seed_proportion* were then seeded with TB disease, with *probability prop_smearpos_HIV0* becoming smear+ and the rest smear-.

The model was run with a constant population size for 100 years, and then a further 100 years with a growing population size, to allow the population age distribution and TB incidence and mortality to reach equilibrium. At that point, the model was considered to represent the year 2000, and realistic trends in HIV and TB were simulated from that point onwards.

2.4 Model scheduling

The majority of events in the model were simulated using continuous time.

The two exceptions to this were the creation of new people, and the *Mtb* transmission process, which used a monthly time step.

2.5 Model runs and calibration

The model was fitted by hand, by varying model input parameters until the model gave an acceptable fit to the fitting targets.

The model was run 2000 times for each fitted scenario and intervention, with the results averaged over the 2000 runs. Model outputs were outputted annually, giving mid-year values for cross-sectional count outputs, and end of year values for cumulative count outputs.

2.6 Demography

Individuals were introduced into the model at age 15. People aged <15 were not modelled, as the risk of *Mtb* transmission from children is low[2], and contact data were not available from children from the study population.

During the initial run-in period, a constant population size of 10,000 was simulated. Each month, the number of people alive in the model was counted, and additional people created to restore the model population size to 10,000. After the initial run-in period, a constant birth rate per person alive was simulated, with the number of new people to be created each month equal to binomial(population size, *birth_rate*).

Exact age was tracked for each simulated individual; however, individuals were grouped into three age groups, 15-29 years, 30-49 years, and 50-79 years. A number of parameter values in the model varied by age group and sex: background mortality rates, HIV seeding proportions, HIV infection rates, and contact rates in clinics and 'other' locations.

There were four types of mortality in the model

- HIV mortality
- TB mortality
- Background mortality
- All individuals die upon reaching the age of 80 years

The background morality rates varied by age and sex, and were constant over time within each age group. TB and HIV mortality are described in the sections on TB and HIV.

2.6.1 Fitting targets

The model was fitted to provincial-level data from KwaZulu-Natal on the estimated growth in population size between 2015 and 2019, the proportion of the population who are male in 2018, and the proportion of men and women in each of the three simulated age groups, by varying the simulated birth rate and age and sex specific background mortality rates. As in- and out-migration were not explicitly simulated, the background mortality rates were not designed to accurately reflect true (non-HIV and non-TB) mortality rates by age, but instead to also incorporate the effects of in- and out-migration on the population age distribution.

2.7 Social contact

Three types of social contact were simulated in the model: contact between household members, contact occurring in clinics, and contact occurring in all other locations.

McCreesh N, *et al. BMJ Global Health* 2022; 7:e007136. doi: 10.1136/bmjgh-2021-007136

2.7.1 Household members

In the model, it was assumed that each individual has *contact_time_each_hh_mem* = 572 hours of indoor contact with each member of their household each month (18.8 hours per day * 365.25 days / 12 months).

2.7.2 Clinics

In line with the empirical data, the rate of clinic visiting in the model varied by sex and HIV/ART status, but not by age group. For each sex and HIV/ART status strata, 50% of the simulated population was assumed to be in a high clinic visiting group, and 50% in a low clinic visiting group. Clinic visiting rates in each group, for each strata, were determined by fitting a Poisson distribution to the data on the proportion of people in each strata who visited a clinic 0, 1 ,2-5 or 6+ times in the past six months, and the overall rate of clinic visiting in the strata, using a sum of least squares approach. Individuals changed between the high and low clinic visiting groups every six months with probability *clinic_rate_switch_prob*.

The rate of clinic visiting also varied for individuals with untreated TB disease (in the states smear-positive disease (smear+) and smear-negative disease (smear-)). Compared to individuals of the same sex, HIV/ART strata, and clinic visiting group, the rate of clinic visiting in people with untreated TB disease was increased by a factor of *increased_contact_time_clinics_tb*.

It was assumed in the model that all individuals had 140 adult contact hours on each clinic visit.

Individual clinic visits were not explicitly simulated in the model, instead each individual had a set amount of contact time in clinics each month (e.g. *contact_time_clinic_m_HIV01_low*), equal to the assumed mean number of clinic visits in a month (by sex, HIV/ART status, and clinic visiting group) multiplied by the mean contact time per visit.

2.7.3 Other locations

Mean contact time in other locations in the model varied by sex, age group, and HIV/ART status, with mean contact time by group (e.g. *contact_time_other_m_age0_HIV01*) estimated using a regression model containing sex, age group, and HIV/ART status as categorical variables.

2.7.4 Fitting targets

increased_contact_time_clinics_tb was varied to fit the model to empirical data from the study community in 2019 on the ratio of estimated prevalence of TB in clinic attendees relative to the general population[3]. The ratio was calculated from the model output as the proportion of all contact time in clinics in the model that was by people with smear+ or smear- TB, divided by the prevalence of smear+ or smear- TB in the whole model population, at the end of June 2019.

2.8 Ventilation

Empirical data on ventilation rates in people's home in rural KwaZulu-Natal suggest mean absolute ventilation rates range from 110-274m^3h^{-1} with windows and doors closed, 457-476 m^3h^{-1} with windows open only, and 988-1187 m^3h^{-1} with windows and doors open[4]. Empirical data from clinic waiting areas show large amount of variation in ventilation rates between different spaces, but they suggest that clinic spaces are generally better ventilated on average than people's homes[5]. We assumed in the model that the rate of transmission from a person with TB disease to a person without is 2.8 times higher in homes than in clinics. As the model is calibrated to an estimate of the proportion of disease that results from transmission between household members, however, the assumption made about ventilation rates in homes vs other spaces has little effect on the results (see Section 2.9.8).

Limited data were available on ventilation rates from other types of location, and showed large amounts of variation[6]. Nevertheless, rates for most locations were more in line with the higher ventilation rates found in clinic waiting areas than the lower rates found in people's houses. For this reason, we assumed in the main scenario in the model that the rate of transmission between a person with TB disease and a person without is the same in other locations as in clinics.

The effects of the assumptions made about ventilation rates in clinics and other locations were explored in a sensitivity analysis (See section 2.14 Uncertainty analysis).

2.9 Tuberculosis

2.9.1 Disease states

Each individual in the model was in one of five main TB states (uninfected, latent, smear+ disease, smear- disease, on treatment), with the latent infection state subdivided by time since infection (Figure 1).

2.9.2 Drug resistance

Tuberculosis was simulated as non-multidrug resistant (non-MDR-TB) or multidrug resistant (MDR-TB). MDR-TB was seeded into the model in 2010 (*introduce_mdr_year*) by making simulated people in the model with *Mtb* infections (latent or active) set their resistance type to MDR-TB with probability *tb_seed_proportion_mdr*. MDR-TB was not introduced into the model earlier to prevent extinction of the strain when the model population size was lower.

Resistance type in the model effected the TB treatment duration. The treatment duration for non-MDR-TB was always six months. For MDR-TB, it was 24 months for all people starting TB treatment before 2016, then 24 months with probability 0.3, and 11 months with probability 0.7[7 8].

TB treatment drop-out rates in the model also varied by resistance type (see Treatment).

2.9.3 Disease progression

The rate of developing tuberculosis disease following infection depended on an individual's time since infection with *Mtb* and their HIV/ART status. For HIV- and HIV+ART+ people, the rate was highest in the first year, falling each year over the subsequent five years, and then lowest from five years following infection. For HIV+ART- people, the rate was highest in the first year following infection, and lower in all subsequent years.

The rate of developing disease also depended on the model year, being reduced by a factor of *decreased_tb_rates_late* for all simulated people in *change_TB_parameters_year* (see section changes in TB parameters over time), and for HIV+ART- people in *change_HIV1_parameters_year* (see section changes in HIV parameters over time).

Upon developing disease, HIV-, HIV+ART-, and HIV+ART+ people developed smear+ disease with *probability prop_smearpos_HIV0*, *prop_smearpos_HIV1*, and *prop_smearpos_HIV2* respectively. All other individuals developed smear- disease.

HIV-, HIV+ART-, and HIV+ART+ people with TB disease self-cured at rate *self_cure_rate_HIV0*, *self_cure_rate_HIV1*, and *self_cure_rate_HIV2* respectively. Upon self-cure, individuals re-entered the latent stage, resetting their time since infection back to zero.

2.9.4 Treatment

Individuals with TB started treatment each month with probability *treatment_rate_HIV0* if HIV-, and *treatment_rate_HIV12* if HIV+. These rates took the value *treatment_rate_HIV0_early* and *treatment_rate_HIV12_early respectively* before *treatment_rate_change_year*, and *treatment_rate_HIV0_late* and *treatment_rate_HIV12_late* respectively afterwards.

After the year that ART was first introduced into the model, *ART_intro_year*, upon starting TB treatment, all HIV+ART- people became HIV+ART+.

Treatment lasted for *treatment_duration_DS* months if non-MDR-TB, and *treatment_duration_MDR* months if MDR-TB. Individuals successfully finishing treatment re-entered the latent stage. Upon doing so, they reset their time since infection back to zero, reflecting the high rates of disease recurrence following treatment[9 10].

Individuals receiving TB treatment dropped out of treatment each month with probability *TB_treatment_dropout_rate_ DS* if they had non-MDR-TB and, *TB_treatment_dropout_rate_MDR* if they had MDR-TB. Upon dropping out of treatment, they returned to active TB disease, with the

same strain of disease (non-MDR-TB or MDR-TB). Different TB treatment drop out rates by HIV status were not simulated, as empirical data showed little difference in treatment success by HIV status in South Africa[11].

2.9.5 Mortality

TB mortality rates in the model depended on disease type (smear- or smear+), HIV/ART status, and whether someone was receiving treatment or not.

Among people not on treatment, the annual TB mortality rate was *TB_mortality_rate_smearpos_HIV0* (*TB_mortality_rate_smearneg_HIV0*) for HIV- with smear+ (smear-) disease, *TB_mortality_rate_smearpos_HIV1* (*TB_mortality_rate_smearneg_HIV1*) for HIV+ART- people with smear+ (smear-) disease, and *TB_mortality_rate_smearpos_HIV2* (*TB_mortality_rate_smearneg_HIV2*) for HIV+ART+ people with smear+ (smear-) disease.

When on treatment, the annual TB mortality rate was *TB_mortality_rate_treatment_DS* for people with non-MDR-TB, and *TB_mortality_rate_treatment* for people with MDR-TB. Different TB mortality rates by HIV status while on TB treatment were not simulated, as empirical data showed little difference in treatment success by HIV status in South Africa[11].

2.9.6 Prevalence of infection in 15-year olds

In 2013, 14.4% of 6-8 year olds were found to be infected with *Mtb* or to be on TB treatment in KwaZulu-Natal, giving an estimated annual rate of infection rate 2.1%[12]. Adjusting by reductions in estimated TB incidence between 2013 and 2018, and by increases in attack rates between childhood and adolescence[13], we estimated that around 24.2% of adolescents in KwaZulu-Natal in 2018 were infected with *Mtb*. Upon being created at the age of 15 years, people in the model therefore set their state to latent with probability 0.242. The remaining people were assumed to be uninfected.

In calculating rates of progression to active disease in individuals with *Mtb* infections at the point of their creation at age 15 in the model, we assigned them a time of infection, *time_of_infection*, from a uniform distribution covering the 15 years before their creation. Their rate of disease progression was then calculated using the same method as was used for people infected at ages >15 years. Progression to disease that occurred prior to the age of 15 was not included in the model.

time_of_infection was also used to determine, *prob_MDR_at_15*, the time-varying probability that individuals with existing infection at age 15 were infected with MDR *Mtb*. *prob_MDR_at_15* was set equal the proportion of the overall force of infection that was from individuals with MDR-TB at their assigned time of infection. For individuals created with a *time_of_infection* between

introduce_mdr_year – 15 and *introduce_mdr_year*, *prob_MDR_at_15* was set equal to *tb_seed_proportion_mdr*.

2.9.7 Changes in TB natural history parameters over time

To reflect secular trends not captured by other time varying parameters in the model (for instance, improvements in nutrition and housing), a step change was modelled in *TB_parameter_change_year*. In *TB_parameter_change_year*, the simulated rate of *Mtb* transmission (*transmission_prob*), and the simulated rates of progression to TB disease following infection were reduced by a factor of *decreased_tb_rates_late*.

2.9.8 Fitting targets

The model was fitted to a range of TB incidence, mortality, and treatment outcome estimates (see section 'Modelling fitting targets').

The model was also fitted to the central value of a range of estimates for the proportion of disease that results from transmission between household members in sub-Saharan African countries[14]. This was done by varying the degree of individual variation in infectiousness between people with tuberculosis, with higher levels of variation leading to a lower proportion of disease resulting from transmission between household members.

2.10 *Mtb* transmission

Mtb transmission in the model was scheduled on a monthly time step. Three transmission 'locations' were simulated, with transmission in each location simulated in turn each month: transmission between household members, transmission in clinics, and transmission in other indoor locations (including transport). Random mixing was assumed in clinics and in other locations.

In all locations, the parameter *transmission_prob* determined the baseline probability of transmission per minute contact between each uninfected or latent person and each person with smear+ or smear- TB. *transmission_prob* took the value *transmission_prob_early* before *TB_parameter_change_year*, and *transmission_prob_early * decreased_tb_rates_late* afterwards.

The baseline *transmission_prob* was then adjusted for a number of factors:

- The simulated ventilation level in the location. The effect of ventilation levels on the rate of transmission is described in the section 'Ventilation'.
- The smear status of the person with TB. We assumed that people with smear- disease are 78% less infectious than people with smear+ TB[15].

- Whether the exposed person was uninfected or latent, and their HIV/ART status. We assumed that latent infection provides 72% protection against reinfection in HIV- people[16], with lower levels of protection in HIV+ART- people, and intermediate levels of protection in HIV+ART+ people.
- The individual-level infectiousness of the person with TB (household transmission only) (see section ‘Individual-level variation in infectiousness).

2.10.1 Individual-level variation in infectiousness

Individuals in the model had an individual level of infectiousness, *infectiousness*. This was sampled at birth for each simulated person from a gamma distribution with mean = 1 and variance = *infectiousness_var*.

The *infectiousness* parameter was assumed to incorporate the effects of all factors that have an effect on the infectiousness of a person with TB, with the exception of whether the disease is smear+ or smear-.

Individual-level variation in infectiousness was simulated when determining *Mtb* transmission between household members, because the variation acts to reduce the rate of transmission between highly regular contacts such as household members, through increasing the effects of saturation[14]. Not incorporating this variation would therefore have resulted in an unrealistically high proportion of disease in the model coming from transmission between household members.

Individual-level variation in infectiousness was not used in the model when determining *Mtb* transmission in clinics and other locations. Instead, the overall mean value of *infectiousness*, 1, was used for all people. This reduced model stochasticity, speeding up the model fitting process, and meaning that far fewer model runs needed to be done per final scenario and intervention. As random mixing was simulated in both clinics and other locations, this had no effect on the average proportion of disease that results from transmission in clinics and other locations in the model.

2.10.2 Household members

To simulate transmission between household members, the number of people with smear+ non-MDR-TB and MDR-TB, and smear- non-MDR-TB and MDR-TB, in each household were counted (N_{sr}, where s=0 indicates smear- disease and s=1 indicates smear+ disease, and where r=0 indicates non-MDR-TB and r=1 indicates MRD-TB), and the mean value of *infectiousness* in household members with smear+ non-MDR-TB and MDR-TB and smear- non-MDR-TB and MDR-TB was calculated for

each household (I_{sr}). If no household members had the corresponding type of disease, then I_{sr} was set to zero.

For each susceptible or latent individual in the household, the probability of infection each month was calculated as:

$$1 - \prod_{s=0}^{1} \prod_{r=0}^{1} (1 - transmission\_prob \times I_{sr} \times ventilation\_weight\_home \times reinfection\_relative\_risk \times W_s)^{N_{sr} * contact\_time\_each\_hh\_mem})$$

Where:

- *reinfection_relative_risk* = 1 if the individual was uninfected, *reinfection_relative_risk_HIV0* if they were HIV- and latently infected, *reinfection_relative_risk_HIV1* if they were HIV+ART- and latently infected, and *reinfection_relative_risk_HIV2* if they were HIV+ART+ and latently infected
- W_s = 1 when s = 1, and W_s = *reduced_transmission_smearneg* when s = 0

The probability that people infected with *Mtb* from transmission from a household member were infected with an MDR strain was calculated as:

$$(\sum_{s=0}^{1} N_{s1} \times I_{s1} \times W_s)/(\sum_{s=0}^{1}\sum_{r=0}^{1} N_{sr} \times I_{sr} \times W_s)$$

2.10.3 Clinics

Each month, the total contact number of people in each class was counted, with class defined as the 60 strata generated by all combinations of:

- Sex (male, female)
- HIV/ART status (HIV-, HIV+ART-, HIV+ART+)
- Clinic visiting group (high, low)
- TB status (smear+ non-MDR-TB, smear- non-MDR-TB, smear+ MDR-TB, smear- MDR-TB, non-infectious (all other TB states))

The total contact time in clinics by people in each class was then calculated, by multiplying the number of people by the mean contact time per person. For people with smear+ and smear- TB, mean contact time was higher by a factor of *increased_contact_time_clinics_tb*, compared to other people in the same sex, HIV/ART, and clinic visiting strata.

Finally, the proportion of all contact time in clinics that were with someone with smear+ non-MDR-TB, smear+ MRD-TB, smear- non-MDR-TB, and smear- MRD-TB was calculated (P_{sr}, where s=0

indicates smear- disease and s=1 indicates smear+ disease, and where r=0 indicates non-MDR-TB and r=1 indicates MRD-TB).

For each susceptible or latent individual in the model, the probability of infection each month from transmission in clinics was then calculated as:

$$1-\prod_{s=0}^{1}\prod_{r=0}^{1}(1-transmission\_prob \times int\_RR\_trans\_clinics \times ventilation\_weight\_clinics \times reinfection\_relative\_risk \times W_s)^{P_{sr} * contact\_time\_clinics \times int\_RR\_contact\_clinics})$$

Where:

- *reinfection_relative_risk* = 1 if the individual was uninfected, *reinfection_relative_risk_HIV0* if they wer HIV- and latently infected, *reinfection_relative_risk_HIV1* if they were HIV+ART- and latently infected, and *reinfection_relative_risk_HIV2* if they were HIV+ART+ and latently infected.
- W_s = 1 when s = 1, and W_s = *reduced_transmission_smearneg* when s = 0.
- *contact_time_clinics* was equal to the mean monthly contact time in clinics for someone of the individual's class.
- *int_RR_trans_clinics* = 1 until 2021 in all scenarios, and took different values from then in some intervention scenarios (see 'Interventions').
- *int_RR_contact_clinics* = *int_RR_contact_clinics_HIV01* if the individual was HIV- or HIV+ART-, and *int_RR_contact_clinics* = *int_RR_contact_clinics_HIV2* if the individual was HIV+ART+. *int_RR_contact_clinics_HIV01* = *int_RR_contact_clinics_HIV2* = 1 until 2021 in all scenarios, and took different values from then in some intervention scenarios (see 'Interventions').
- 1 until 2021 in all scenarios, and took different values from then in some intervention scenarios (see 'Interventions').

The probability that people infected with *Mtb* from transmission in clinics were infected with an MDR strain was calculated as:

$$(\sum_{s=0}^{1} P_{s1} \times W_s)/(\sum_{s=0}^{1}\sum_{r=0}^{1} P_{sr} \times W_s)$$

2.10.4 Other locations

Each month, the total contact number of people in each class was counted, with class is defined as the 90 strata generated by all combinations of:

McCreesh N, *et al. BMJ Global Health* 2022; 7:e007136. doi: 10.1136/bmjgh-2021-007136

- Sex (male, female)
- HIV/ART status (HIV-, HIV+ART-, HIV+ART+)
- Age group (15-29, 30-49, 50-79)
- TB status (smear+ non-MDR-TB, smear- non-MDR-TB, smear+ MDR-TB, smear- MDR-TB, non-infectious (all other TB states))

The total contact time in other location by people in each class was then calculated, by multiplying the number of people by the mean contact time per person.

Finally, the proportion of all contact time in other locations that was with someone with smear+ non-MDR-TB, smear+ MRD-TB, smear- non-MDR-TB, and smear- MRD-TB was calculated (P_{sr}, where s=0 indicates smear- disease and s=1 indicates smear+ disease, and where r=0 indicates non-MDR-TB and r=1 indicates MRD-TB).

For each susceptible or latent individual in the model, the probability of infection each month from transmission in other locations was then calculated as:

$$1 - \prod_{s=0}^{1} \prod_{r=0}^{1} (1 - transmission\_prob \times ventilation\_weight\_other \times reinfection\_relative\_risk \times W_s)^{P_{sr} * contact\_time\_other})$$

Where:

- *reinfection_relative_risk* = 1 if the individual was uninfected, *reinfection_relative_risk_HIV0* if they were HIV- and latently infected, *reinfection_relative_risk_HIV1* if they were HIV+ART- and latently infected, and *reinfection_relative_risk_HIV2* if they were HIV+ART+ and latently infected.
- W_s = 1 when s = 1, and W_s = *reduced_transmission_smearneg* when s = 0
- *contact_time_other* was equal to the mean monthly contact time in other locations for someone of the individual’s class.

The probability that people infected with *Mtb* from transmission in other locations were infected with an MDR strain was calculated as:

$$(\sum_{s=0}^{1} P_{s1} \times W_s)/(\sum_{s=0}^{1} \sum_{r=0}^{1} P_{sr} \times W_s)$$

2.11 HIV/ART

Three HIV states were simulated in the model: HIV-, HIV+ART-, and HIV+ART+.

HIV was introduced into the model in 2000, by seeding a set proportion of each age group and sex at random with HIV. People created in the model at age 15 years were all HIV-. From the introduction of HIV in the model in 2000, HIV- people became HIV+ART- at a rate that varied by age group and sex.

To capture changes in estimated and projected HIV prevalence over time, the value of the HIV incidence parameters for each age group and sex changed twice in the model, in *HIV_inc_change_year1* and *HIV_inc_change_year2*.

ART was introduced in the model in 2005. From the introduction of ART, HIV+ART- people became HIV+ART+ at a rate that varied by sex. To capture changes in estimated ART coverage over time, the values of the ART start rates in the model were changed in *ART_start_rate_change_year*.

From *ART_start_rate_change_year*, all HIV+ART- people starting TB treatment were made HIV+ART+.

HIV mortality was simulated as a constant rate of (non-TB) HIV-related mortality for all HIV+ART- people (*HIV1_mortality_rate*), and all HIV+ART+ people (*HIV2_mortality_rate*),

2.11.1 Effects on TB

HIV and ART status effected a number of TB-related rates and probabilities in the model:

- TB mortality rates
- Rates of progression to disease
- Self-cure rates
- Protection against reinfection from being latently infected
- Probability of developing smear+ disease
- Contact rates

The effects of HIV and ART on parameter values are described in more details in the relevant sections, and the parameter ranges shown in the section ‘Input parameters’. When someone became HIV+ or started ART, the values of all of their rates change immediately.

2.11.2 Changes in HIV parameters over time

CD4 counts for HIV+ART- people were not explicitly simulated, with HIV+ART- being simulated as a single, homogenous group, varying only with age group and sex. As ART coverage increased over time in South Africa, however, the average CD4 count of people not on ART is likely to have risen, and the impact on TB natural history of being HIV+ART- is likely to changed.

McCreesh N, *et al. BMJ Global Health* 2022; 7:e007136. doi: 10.1136/bmjgh-2021-007136

To allow the effects of increased ART coverage on TB to be adequately captured in the model, enabling the model to be fitted to trends in TB incidence over time, a step change in the values of certain HIV related parameters was simulated, starting in *change_HIV1_parameters_year*.

From *change_HIV1_parameters_year*, the degree of protection that latent infection gave against reinfection in HIV+ART- people, *reinfection_relative_risk_HIV1*, was increased from *reinfection_relative_risk_HIV1_early* to *reinfection_relative_risk_HIV1_late*, and the rate of developing disease in more than one year following infection in HIV+ART- people was decreased from *develop_tb_reactivation_rate_HIV1_early* to *develop_tb_reactivation_rate_HIV1_late*. From *TB_parameter_change_year*, these rates were also decreased by *decreased_tb_rates_late* (see section 'Changes in TB natural history parameters over time' and Table S6). As the rate of developing disease in the first year following infection in HIV+ART- people was calculated relative to the rate in subsequent years in the model, this also decreased the rate in the first year following infection.

Order of two parameter value change years	First time period		Second time period		Third time period	
	Time range	Parameter value	Time range	Parameter value	Time range	Parameter value
change_hiv1_parameters_year < *TB_parameter_change_year*	Start to *change_hiv1_parameters_year*	*reinfection_relative_risk_HIV1_early*	*change_hiv1_parameters_year* to *TB_parameter_change_year*	*reinfection_relative_risk_HIV1_late*	*TB_parameter_change_year* to end	*reinfection_relative_risk_HIV1_late* * *decreased_tb_rates_late*
change_hiv1_parameters_year > *TB_parameter_change_year*	Start to *TB_parameter_change_year*	*reinfection_relative_risk_HIV1_early*	*TB_parameter_change_year* to *change_hiv1_parameters_year*	*reinfection_relative_risk_HIV1_early* * *decreased_tb_rates_late*	*change_hiv1_parameters_year* to end	*reinfection_relative_risk_HIV1_late* * *decreased_tb_rates_late*
change_hiv1_parameters_year = *TB_parameter_change_year*	Start to *change_hiv1_parameters_year*/ *TB_parameter_change_year*	*reinfection_relative_risk_HIV1_early*	*change_hiv1_parameters_year*/ *TB_parameter_change_year* to end	*reinfection_relative_risk_HIV1_late* * *decreased_tb_rates_late*	NA	NA

Table S6. Value taken by the model parameter *reinfection_relative_risk_HIV1*, over time, depending on the relative values of the parameter *change_hiv1_parameters_year* and *TB_parameter_change_year*.

2.11.3 Fitting targets

The model was fitted to a range of HIV prevalence and ART coverage targets, based on empirical estimates from the study population[17]. These are described in full in the section 'Fitting targets'.

In addition to this, the model was fitted to estimated future trends in HIV prevalence and ART coverage by sex, from provincial HIV model (Thembisa) estimates[18 19]. As the Thembisa estimates were for the province as a whole, and the model was fitted to historic trends from the study population, the model was fitted to estimates *changes* in HIV prevalence and ART coverage by sex between 2020 and 2030, rather than the absolute estimates.

2.12 Interventions

Seven potential infection control interventions had been identified in qualitative research and system dynamics modelling exercises conducted as part of the *Umoya omuhle* project[20]. The effect of the interventions on patient contacts and infection risk in clinics were estimated in previous modelling work, using a within-clinics model that simulated the flow of patients through clinics, and ventilation rates and infection risk in clinic waiting areas[21]. The interventions were:

1) **Opening windows and doors.** Ensuring windows and doors in waiting areas are kept open at all times. This was implemented in the within-clinics model through increasing simulated ventilation rates
2) **Simple clinic retrofits.** Building retrofits are changes to the building to improve ventilation rates. This could include installing lattice brickwork or whirlybird fans. Due to the large amount of variation between clinic spaces in the types of building retrofits that would be suitable, and the lack of sufficient data on the effects of the retrofits on ventilation rates in different types of spaces, we did not model specific retrofits or packages of retrofits. Instead, in the within clinics model, we simulated an undefined package of retrofits that are sufficient to increase air changes per hour to a minimum of 12 in all rooms, chosen in line with WHO guidelines[22 23]
3) **Ultraviolet Germicidal Irradiation (UVGI) system.** We assumed in this intervention that appropriate and well maintained UVGI systems are installed in all indoor clinic waiting areas. This was implemented in the within-clinics model through an additional quanta clearance rate, equivalent to a ventilation rate of 24 ACH (95% CI 9.9-62)[24].
4) **Surgical mask wearing by patients.** We simulated a scenario where 70% of patients wear surgical masks 90% of the time. Masks were assumed in the within-clinics model to reduce the rate of quanta production by 75% (95% CI 56-85%)[25], and have no effect on rate of infection for the person wearing the mask[26].

5) **Increasing CCMDD coverage.** South Africa's Central Chronic Medicine Dispensing and Distribution (CCMDD) programme is designed to allow patients with stable chronic health conditions to collect their medicines from convenient locations, such as local pharmacies[27]. This means that they do not need to queue at clinics unnecessarily. The purpose of this intervention was to increase the utilisation of CCMDD and similar programmes by eligible patients, and to ensure that pick-up points do not require patients to queue at clinics. We assumed that 92% (95% CI 84-95%) of patients could have their ART appointments reduced to once every 6 months[28], and that the remaining 8% of people need monthly ART appointments. This was implemented in the within-clinics model through removing 31% (IQR 22-34%) of ART patients, chosen at random each model run.
6) **Queue management system with outdoor waiting areas.** Empirical data show that clinic waiting areas are often crowded, and that in many clinics patients wait in unsuitable areas such as corridors[29]. This is partly due to patient concerns that if they wait in other areas, they may not hear their name being called, and may miss their turn. This intervention therefore combined a large, covered outdoor waiting area with a queue management system, such as numbered tickets or an electronic tracking system. We assumed in the within-clinics model that only a small number of patients were allowed to wait inside the clinic, with the rest waiting in a large, covered, outdoor waiting area, with a very high ventilation rate of 52-70 ACH[30].
7) **Appointment systems.** In this intervention, we simulated a date-time appointment system to reduce clinic overcrowding, through spacing out the arrival times of patients in the within-clinics model.

The estimated effects of the interventions on patient contacts and infection risk in clinics from the within-clinics model were used to parameterise the effects of the interventions in this model, allowing their wider effects on community-level disease incidence to be estimated. The interventions were implemented through changing parameter values, starting in 2021 (see Table S7).

The 'best estimates' of intervention effects in this model were informed by the median impacts from the within-clinics model. The minimum and maximum estimates were informed by the interquartile ranges from the within-clinics model. The interquartile range was used, rather than the full range, as the most extreme effects from the within-clinics model were assumed to reflect day to day variation, rather than genuine uncertainty in intervention effects.

McCreesh N, *et al. BMJ Global Health* 2022; 7:e007136. doi: 10.1136/bmjgh-2021-007136

Intervention	Parameters changed	Parameter description	Simulated value (from 2021)		
			**Minimum effect**	**Best estimate**	**Maximum effect**
**Windows and doors**	*int_RR_trans_clinics*	Modifier of risk of infection per minute contact occurring in clinics	0.75	0.45	0.28
**Retrofits**	*int_RR_trans_clinics*		0.84	0.55	0.36
**UVGI**	*int_RR_trans_clinics*		0.36	0.23	0.15
**Masks**	*int_RR_trans_clinics*		0.58	0.53	0.50
**Queue management and outdoor waiting area**	*int_RR_trans_clinics*		0.24	0.17	0.12
**CCMDD**	*int_RR_contact_clinics_HIV2*<br>*int_RR_contact_clinics_HIV01*	Modifier of mean contact hours in clinics for people who are HIV+ART+ and HIV- or HIV+ART- respectively	HIV2: 0.91<br>HIV01: 0.92	HIV2: 0.72<br>HIV01: 0.87	HIV2: 0.58<br>HIV01: 0.81
**Appointment system**	*int_RR_contact_clinics_HIV2*<br>*int_RR_contact_clinics_HIV01*	Modifier of mean contact hours in clinics for people who are HIV+ART+ and HIV- or HIV+ART- respectively	Both: 0.55	Both: 0.38	Both: 0.25

Table S7. Simulated intervention effects. Parameters changed in each intervention, and the values simulated

2.13 Results calculations

When calculating the proportion of disease that resulted from transmission in clinics in the model, simulated individuals who developed disease from an infection that occurred before the age of 15 years were not included, as their location of infection could not be determined.

Intervention effects on TB incidence and mortality were calculated as relative changes in rates, compared to a scenario where no interventions are simulated. As the simulated proportion of people created in the model at age 15 years who had a latent infection is constant over time, simulated individuals who developed or died from TB disease from an infection that occurred before the age of 15 years were not included when estimating intervention effects on TB incidence and mortality.

2.14 Uncertainty analyses

A number of univariate sensitivity analyses were conducted:

- **Proportion of outside-household contact time occurring in clinics (clinic contact time).** From the social contact data, overall, we estimated that 5.3% (95% CI 2.8-8.0%) of contact time that occurs outside respondents' own homes occurs in clinics (weighted to model population size by sex and HIV/ART status in 2019). In the sensitivity analysis, we explored the effect of multiplying all of the clinic contact parameters by 0.53 (=2.8/5.3) and 1.51 (=8.0/5.3). The simulated clinic contact times are shown in Table S8.
- **Prevalence of TB in clinic attendees relative to the general population (TB in clinics).** In the main scenario, the model was fitted to a prevalence of TB in clinic attendees relative to the community prevalence of 1.86. In the sensitivity analysis, the model is fit to the upper bounds of the empirical 95% confidence interval (1.1-3.1)[3]. Fitting to the lower bound would have required the value of *increased_contact_time_clinics_tb* to be less than one. In other words, it would have required simulating a lower rate of clinic visiting in people with TB compared to people without, controlling for sex and HIV/ART status. This was considered to be implausible, therefore *increased_contact_time_clinics_tb* = 1 was used as the lower bound.
- **Proportion of disease from household transmission (Household transmission).** In the main scenario, we fitted the model to 13.5% of disease resulting from transmission between household members. In the sensitivity analysis, the model was fitted to 8% and 19%[14] of disease resulting from transmission between household members. This was achieved primarily by changing the value of *infectiousness_var*.

- **Ventilation rates in clinics (Clinic ventilation).** In the main scenario, mean ventilation rates were assumed to be the same in clinics as in other locations, with *ventilation_weight_clinic* = *ventilation_weight_other* = 1. In the sensitivity analysis, the value of *ventilation_weight_clinic* was changed to 0.5 and to 2.
- **Movement between high and low clinic visiting groups (Clinic risk groups).** As the social contact survey collected data on number of clinics visits over a six-month period only, we were unable to distinguish the extent that differences in clinic visiting rates between people of the same sex and HIV/ART status were due to long-term, stable differences vs shorter term fluctuations in clinic use. In the main scenario, we simulated people switching between clinic visiting risk groups every six months with probability *clinic_rate_switch_prob* = 0.25. In the sensitivity analysis, we simulated people switching with probability 0 and 0.5.
- **Clinic visiting rates by HIV+ART- people, relative to HIV- people (HIV+ART- clinic visiting).** In the social contact data collection, only 13 people reported being HIV+ART-. In addition, HIV-status was self-reported, and we could therefore not accurately distinguish between HIV- and undiagnosed HIV+ people, particularly when the reported date of the last HIV-test was not recent. We therefore had no empirical data on rates of clinic visiting in HIV+ART- people. In the main scenario, we assumed that the rates are the same in HIV+ART- and in HIV- people, and determined the rates from the empirical data for all people who did not report being on ART. In the sensitivity analysis, we assumed that rates in HIV+ART- people are half that of HIV- people, and that rates in HIV+ART- people are the same as for HIV+ART+ people. In both scenarios, we also adjusted the HIV- clinic visiting rates to keep the overall mean clinic visiting rates in 2020 for HIV+ART- and HIV- people constant. The simulated clinic contact times are shown in Table S8.
- **Future HIV incidence.** Estimated future trends in HIV incidence were taken from the projections from a provincial-level HIV model, Thembisa[18 19], with the model fitted to the estimated change in HIV prevalence in men and women between 2020 and 2030. While Thembisa did provide 95% limits for its estimates, we considered them to be unrealistically narrow. For instance, the 95% limits for the projected prevalence of HIV in men aged 15-49 in 2030 was 11.4-12.3%. In the sensitivity analysis, we therefore chose to simulate relative changes in HIV incidence by sex from 2020 compared to the preceding time period, that were 50% lower and 150% higher than the simulated changes in the main scenario.

In all sensitivity analyses, the model was recalibrated to the same fitting targets (with the exception of the targets explicitly changed in the sensitivity analysis).

	Best	Clinic contact time		HIV+ART- clinic visiting	
		Low	High	Low	High
contact_time_clinic_m_HIV0_low	493	256	737	520	143
contact_time_clinic_m_HIV1_low	493	256	737	260	3468
contact_time_clinic_m_HIV2_low	3468	1803	5185	3468	3468
contact_time_clinic_f_HIV0_low	2322	1207	3472	2483	1435
contact_time_clinic_f_HIV1_low	2322	1207	3472	1242	8276
contact_time_clinic_f_HIV2_low	8276	4302	12374	8276	8276
contact_time_clinic_m_HIV0_high	5507	2863	8234	5812	5167
contact_time_clinic_m_HIV1_high	5507	2863	8234	2906	8400
contact_time_clinic_m_HIV2_high	8400	4367	12560	8400	8400
contact_time_clinic_f_HIV0_high	8609	4475	12872	9206	8658
contact_time_clinic_f_HIV1_high	8609	4475	12872	4603	8276
contact_time_clinic_f_HIV2_high	8276	4302	12374	8276	8276

Table S8. Simulated clinic contact time per month in the best scenario, and clinic contact time and HIV+ART- clinic visiting scenarios. Values in all other uncertainty analysis scenarios are the same as in the best scenario.

2.15 Input parameters

Name	Description	Value/range	Source
	**Tuberculosis parameters**		
tb_seed_proportion	Proportion of people seeded with TB at the start of the model run	0.005	NA. Model allowed to reach equilibrium before output produced
infection_seed_proportion	Proportion of people seeded with latent Mtb infection at the start of the model run	0.7	NA. Model allowed to reach equilibrium before output produced
transmission_prob_early	Baseline rate of *Mtb* transmission per minute meeting time (before adjustment)	0-1	Varied to fit data
TB_parameter_change_year	Year from which the value of *transmission_prob* and simulated disease progression rates are changed	2007-2018	From year in which estimated TB incidence starts to decline, to final TB incidence fitting year
decreased_tb_rates_late	Multiplier for transmission rate and disease progression rates from *TB_parameter_change_year*	0-1	Varied to fit data
reduced_transmission_smearneg	Lower transmission rate with smear- disease, relative to smear+	0.22	Houben[15]

reinfection_relative_risk_HIV0	Reduced probability of transmission to people with latent infections, relative to uninfected people (HIV-)	0.28	Dowdy and Chaisson[16]
reinfection_relative_risk_HIV1_early	Reduced probability of transmission to people with latent infections, relative to uninfected people, prior to *change_HIV1_parameters_year* (HIV+ART-)	> 0.75	Dowdy and Chaisson[16]
reinfection_relative_risk_HIV1_late	Reduced probability of transmission to people with latent infections, relative to uninfected people, from *change_HIV1_parameters_year* (HIV+ART-)	> *reinfection_relative_risk_HIV2*<br>< 0.75	Dowdy and Chaisson[16]
reinfection_relative_risk_HIV2	Reduced probability of transmission to people with latent infections, relative to uninfected people (HIV+ART+)	> *reinfection_relative_risk_HIV0*<br>< *reinfection_relative_risk_HIV1_late*	
infectiousness_var	The between-individual variance in infectiousness	>0	Varied freely to fit data
self_cure_rate_HIV0	The annual rate of self-cure for HIV- people	0.2	Estimated from Menzies *et al*[31]
self_cure_rate_HIV1	The annual rate of self-cure for HIV+ART-	0.08	Estimated from Menzies *et al*[31]
self_cure_rate_HIV2	The annual rate of self-cure for HIV+ART+	0.14	Estimated from Menzies *et al*[31]
TB_mortality_rate_smearpos_HIV0	Annual rate of mortality from smear+ pulmonary or extrapulmonary TB for HIV- people	0.335-0.449	Ragonnet *et al* (2020)[32]

TB_mortality_rate_smearpos_HIV1	Annual rate of mortality from smear+ pulmonary or extrapulmonary TB for HIV+ART- people	> *TB_mortality_rate_smearpos_HIV0*	
TB_mortality_rate_smearpos_HIV2	Annual rate of mortality from smear+ pulmonary or extrapulmonary TB for HIV+ART+ people	Between 0.16 and 0.91 times *TB_mortality_rate_smearpos_HIV1*, and ≥ *TB_mortality_rate_smearpos_HIV0*	Dheda *et* al (2004)[33] and Lawn *et al* (2009)[34]
TB_mortality_rate_smearneg_HIV0	Annual rate of mortality from smear- pulmonary TB for HIV- people	0.017-0.035	Ragonnet *et al* (2020)[32]
TB_mortality_rate_smearneg_HIV1	Annual rate of mortality from smear- pulmonary TB for HIV+ART-	> *TB_mortality_rate_smearneg_HIV0* and < *TB_mortality_rate_smearpos_HIV1*	
TB_mortality_rate_smearneg_HIV2	Annual rate of mortality from smear- pulmonary TB for HIV+ART+	Between 0.16 and 0.91 times *TB_mortality_rate_smearneg_HIV1*, and ≥ *TB_mortality_rate_smearneg_HIV0*	Dheda *et* al (2004)[33] and Lawn *et al* (2009)[34]
TB_mortality_rate_treatment_DS	Annual TB mortality rate when receiving TB treatment, for DS TB	≥0	Vary freely to fit data on treatment outcomes
TB_mortality_rate_treatment_MDR	Annual TB mortality rate when receiving TB treatment, for MDR TB	≥0	Vary freely to fit data on treatment outcomes

TB_treatment_dropout_rate_DS	Annual rate of dropping out of TB treatment for people with DS TB	≥0	Vary freely to fit data on treatment outcomes
TB_treatment_dropout_rate_MDR	Monthly rate of dropping out of TB treatment for people with MDR TB	≥0	Vary freely to fit data on treatment outcomes
treatment_rate_HIV0_early	Annual rate of starting treatment for HIV- people before *treatment_rate_change_year1*	≥0	Vary freely to fit data
treatment_rate_HIV12_early	Annual rate of starting treatment for HIV+ people before *treatment_rate_change_year1*	≥0	Vary freely to fit data
treatment_rate_HIV0_late	Annual rate of starting treatment for HIV- people from *treatment_rate_change_year1*	≥0	Vary freely to fit data
treatment_rate_HIV12_late	Annual rate of starting treatment for HIV+ people from *treatment_rate_change_year1*	≥0	Vary freely to fit data
treatment_rate_change_year	Year in which the values of the treatment start rate parameters change	2010	Data suggests treatment coverage was relatively stable from 2010[11]
treatment_duration_DS	Length of DS TB treatment in months	6	Managing TB in a New Era of Diagnostics[8]
treatment_duration_MDR	Length of MDR TB treatment in months	24 until 2016, then 30% probability 24, 70% probability 11	Expert opinion, WHO[7], and Managing TB in a New Era of Diagnostics[8]
develop_tb_y1_rate_HIV0	Annual rate of developing TB for HIV- people during the 1st year following infection, before	0.0866	Kasaie *et al*[35]

	TB_parameter_change_year		
develop_tb_y2_rate_HIV0	Annual rate of developing TB for HIV- people during the 2nd year following infection, before *TB_parameter_change_year*	0.0355	Kasaie *et al*[35]
develop_tb_y3_rate_HIV0	Annual rate of developing TB for HIV- people during the 3rd year following infection, before *TB_parameter_change_year*	0.0112	Kasaie *et al*[35]
develop_tb_y4_rate_HIV0	Annual rate of developing TB for HIV- people during the 4th year following infection, before *TB_parameter_change_year*	$7.4 * 10^{-3}$	Kasaie *et al*[35]
develop_tb_y5_rate_HIV0	Annual rate of developing TB for HIV- people during the 5th year following infection, before *B_parameter_change_year*	$2.4 * 10^{-3}$	Kasaie *et al*[35]
develop_tb_reactivation_rate_HIV0	Annual rate of developing TB for HIV- people who have been infected for more than 5 years (late latent), before *TB_parameter_change_year*	$5.0 * 10^{-4}$	Kasaie *et al*[35]
develop_tb_y1_rate_HIV2	Annual rate of developing TB for HIV+ART+ people during the 1st year following infection, before *TB_parameter_change_year*	2 * *develop_tb_y1_ rate_HIV0*	Lawn *et al*[36]
develop_tb_y2_rate_HIV2	Annual rate of developing TB for HIV+ART+ people during the 2nd year following infection, before *TB_parameter_change_year*	2 * *develop_tb_y2_ rate_HIV0*	Lawn *et al*[36]

develop_tb_y3_rate_HIV2	Annual rate of developing TB for HIV+ART+ people during the 3rd year following infection, before *TB_parameter_change_year*	2 * *develop_tb_y3_ rate_HIV0*	Lawn *et al*[36]
develop_tb_y4_rate_HIV2	Annual rate of developing TB for HIV+ART+ people during the 4th year following infection, before *TB_parameter_change_year*	2 * *develop_tb_y4_ rate_HIV0*	Lawn *et al*[36]
develop_tb_y5_rate_HIV2	Annual rate of developing TB for HIV+ART+ people during the 5th year following infection, before *TB_parameter_change_year*	2 * *develop_tb_y5_ rate_HIV0*	Lawn *et al*[36]
develop_tb_reactivation_rate_HIV2	Annual rate of developing TB for HIV+ART+ people who have been infected for more than 5 years (late latent), before *TB_parameter_change_year*	2 * *develop_tb_ reactivation_rate_HIV0*	Lawn *et al*[36]
develop_tb_reactivation_ rate_HIV1_early	Annual rate of developing TB for HIV+ART- people who have been infected for more than 1 year, before *change_hiv1_parameters_year*, and before the adjustment that occurs from *TB_parameter_change_year*	> *develop_tb_reactivation_ rate_HIV2*	
develop_tb_reactivation_ rate_HIV1_late	Annual rate of developing TB for HIV+ART- people who have been infected for more than 1 year, from *change_hiv1_parameters_year* onwards, and before the adjustment that occurs from *TB_parameter_change_year*	> *develop_tb_reactivation_ rate_HIV2* and < *develop_ tb_reactivation_ rate_HIV1_late*	

increased_develop_tb_y1_rate_HIV1	Increased rate of developing TB for HIV+ART- people, during the first year following infection compared to subsequent years	5.14	Dowdy and Chaisson (2009)[16]
prop_smearpos_HIV0	Proportion of HIV- people who develop TB, who develop smear+ disease	0.45	Corbett *et al* (2003)[37]
prop_smearpos_HIV1	Proportion of HIV positive people, not on ART, who develop TB, who develop smear+ disease	0.35	Corbett *et al* (2003)[37]
prop_smearpos_HIV2	Proportion of HIV positive people, on ART, who develop TB, who develop smear+ disease	0.4	Intermediate between HIV- and HIV+ART-
introduce_MDR_year	Year that MDR TB is introduced into the model	2010	Model population size large enough to prevent strain extinction
seed_prop_MDR	Proportion of people with Mtb infections who are seeded with MDR TB	0.029	Ismail *et al* 2018[38]
**HIV parameters**			
HIV_intro_year	Year that HIV is introduced into the model	2000	
hiv_prev_initial_m0	Proportion of males aged 15-29 seeded with HIV at its introduction in *HIV_intro_year*	0.176	2002 HIV prevalence survey[39]
hiv_prev_initial_m1	Proportion of males aged 30-49 seeded with HIV at its introduction in *HIV_intro_year*	0.177	2002 HIV prevalence survey[39]
hiv_prev_initial_m2	Proportion of males aged 50+ seeded with HIV at its introduction in *HIV_intro_year*	0.073	2002 HIV prevalence survey[39]

hiv_prev_initial_f0	Proportion of males aged 15-29 seeded with HIV at its introduction in *HIV_intro_year*	0.105	2002 HIV prevalence survey[39]
hiv_prev_initial_f1	Proportion of males aged 30-49 seeded with HIV at its introduction in *HIV_intro_year*	0.174	2002 HIV prevalence survey[39]
hiv_prev_initial_f2	Proportion of males aged 50+ seeded with HIV at its introduction in *HIV_intro_year*	0.064	2002 HIV prevalence survey[39]
HIV1_mortality_rate	Annual HIV mortality rate in HIV+ART- people	0.1	Mossong *et al* (2013)[40]
HIV2_mortality_rate	Annual HIV mortality rate in HIV+ART+ people	0.0027	Brinkhof *et al* (2009)[41]
hiv_inc_early_f0	Annual HIV incidence rate between *HIV_intro_year* and *HIV_inc_change_year1* in females aged 15-29	0-1	Varied to fit data
hiv_inc_early_f1	Annual HIV incidence rate between *HIV_intro_year* and *HIV_inc_change_year1* in females aged 30-49	0-1	Varied to fit data
hiv_inc_early_f2	Annual HIV incidence rate between *HIV_intro_year* and *HIV_inc_change_year1* in females aged 50-79	0-1	Varied to fit data
hiv_inc_early_m0	Annual HIV incidence rate between *HIV_intro_year* and *HIV_inc_change_year1* in males aged 15-29	0-1	Varied to fit data
hiv_inc_early_m1	Annual HIV incidence rate between *HIV_intro_year* and *HIV_inc_change_year1* in males aged 30-49	0-1	Varied to fit data
hiv_inc_early_m2	Annual HIV incidence rate between *HIV_intro_year* and *HIV_inc_change_year1* in males aged 50-79	0-1	Varied to fit data

hiv_inc_mid_f0	Annual HIV incidence rate between *HIV_inc_change_year1* and *HIV_inc_change_year2* in females aged 15-29	0-1	Varied to fit data
hiv_inc_mid_f1	Annual HIV incidence rate between *HIV_inc_change_year1* and *HIV_inc_change_year2* in females aged 30-49	0-1	Varied to fit data
hiv_inc_mid_f2	Annual HIV incidence rate between *HIV_inc_change_year1* and *HIV_inc_change_year2* in females aged 50+	0-1	Varied to fit data
hiv_inc_mid_m0	Annual HIV incidence rate between *HIV_inc_change_year1* and *HIV_inc_change_year2* in males aged 15-29	0-1	Varied to fit data
hiv_inc_mid_m1	Annual HIV incidence rate between *HIV_inc_change_year1* and *HIV_inc_change_year2* in males aged 30-49	0-1	Varied to fit data
hiv_inc_mid_m2	Annual HIV incidence rate between *HIV_inc_change_year1* and *HIV_inc_change_year2* in males aged 50+	0-1	Varied to fit data
HIV_inc_reduction_late_m	Annual relative change in HIV incidence in males from *HIV_inc_change_year2*, compared to the incidence in the same age group between *HIV_inc_change_year1* and *HIV_inc_change_year2*	0-1	Varied to fit data

HIV_inc_reduction_late_f	Annual relative change in HIV incidence in females from *HIV_inc_change_year2*, compared to the incidence in the same age group between *HIV_inc_change_year1* and *HIV_inc_change_year2*	0-1	Varied to fit data
HIV_inc_change_year1	Year at which HIV incidence parameters change for the first time	2012	Estimated year at which HIV incidence started to decline in the DSA area[17]
HIV_inc_change_year2	Year at which HIV incidence parameters change for the second time	2021	To allow projected future trend in HIV prevalence to be simulated
ART_intro_year	Year that ART is introduced into the model	2005	Coverage of ART was very low in South Africa prior to 2005[42]
ART_start_rate_change_year	Year at which the rate of starting ART changes	2013	Changed year after first ART prevalence fitting target
ART_start_rate_early_m	Annual rate of starting ART for HIV+ males between *ART_intro_year* and *ART_start_rate_change_year*	0-1	Varied to fit data
ART_start_rate_early_f	Annual rate of starting ART for HIV+ females between *ART_intro_year* and *ART_start_rate_change_year*	0-1	Varied to fit data

ART_start_rate_late_m	Annual rate of starting ART for HIV+ males after *ART_start_rate_change_year*	0-1	Varied to fit data
ART_start_rate_late_f	Annual rate of starting ART for HIV+ females after *ART_start_rate_change_year*	0-1	Varied to fit data
change_HIV1_parameters_year	Year at which the values of *reinfection_relative_risk_HIV1* and *develop_tb_reactivation_rate_HIV1* are changed from their 'early' values to their 'late' values	>2005	After the introduction of ART in the model
**Demography parameters**			
initial_pop_size	Initial population size	10000	Balance of model run times and degree of stochasticity in individual runs
initial_m_age0	Initial proportion of males in the age group 15-29	0.432	Same as the desired age distribution in 2018
initial_m_age1	Initial proportion of males in the age group 30-49	0.387	Same as the desired age distribution in 2018
initial_m_age2	Initial proportion of males in the age group 50-79	0.181	Same as the desired age distribution in 2018
initial_f_age0	Initial proportion of females in the age group 15-29	0.382	Same as the desired age distribution in 2018
initial_f_age1	Initial proportion of females in the age group 30-49	0.363	Same as the desired age distribution in 2018

initial_f_age2	Initial proportion of females in the age group 50-79	0.255	Same as the desired age distribution in 2018
birth_rate	Annual birth rate per person	0-1	Varied to fit data
mean_hh_size	Mean simulated household size (individuals aged 15+ years)	3.64	Estimated from empirical data (see section 'Household sizes')
hhsize_parameter_a	See section 'Household size'	0.2	Estimated from empirical data (see section 'Household sizes')
hhsize_parameter_b	See section 'Household size'	4.2	Estimated from empirical data (see section 'Household sizes')
mortality_rate_m_age0	Annual baseline (non-TB or HIV) mortality for males aged 15-29	0-1	Varied to fit data
mortality_rate_m_age1	Annual baseline (non-TB or HIV) mortality for males aged 30-49	0-1	Varied to fit data
mortality_rate_m_age2	Annual baseline (non-TB or HIV) mortality for males aged 50+	0-1	Varied to fit data

mortality_rate_f_age0	Annual baseline (non-TB or HIV) mortality for females aged 15-29	0-1	Varied to fit data
mortality_rate_f_age1	Annual baseline (non-TB or HIV) mortality for females aged 30-49	0-1	Varied to fit data
mortality_rate_f_age2	Annual baseline (non-TB or HIV) mortality for females aged 50+	0-1	Varied to fit data
**Contact time parameters**			
contact_time_each_hh_mem	Minutes of indoor contact time per month between each household member	34328	Social contact survey
contact_time_other_m_age0_HIV01	Minutes of contact time per month in other settings for HIV- males and HIV+ART- males, aged 15-29	138917	Social contact survey
contact_time_other_m_age0_HIV2	Minutes of contact time per month in other settings for HIV+ART+ males, aged 15-29	116328	Social contact survey
contact_time_other_m_age1_HIV01	Minutes of contact time per month in other settings for HIV- males and HIV+ART- males, aged 30-49	98160	Social contact survey
contact_time_other_m_age1_HIV2	Minutes of contact time per month in other settings for HIV+ART+ males, aged 30-49	75571	Social contact survey
contact_time_other_m_age2_HIV01	Minutes of contact time per month in other settings for HIV- males and HIV+ART- males, aged 50+	94046	Social contact survey
contact_time_other_m_age2_HIV2	Minutes of contact time per month in other settings for HIV+ART+ males, aged 50+	71457	Social contact survey

contact_time_other_f_age0_HIV01	Minutes of contact time per month in other settings for HIV- females and HIV+ART- females, aged 15-29	143625	Social contact survey
contact_time_other_f_age0_HIV2	Minutes of contact time per month in other settings for HIV+ART+ females, aged 15-29	121036	Social contact survey
contact_time_other_f_age1_HIV01	Minutes of contact time per month in other settings for HIV- females and HIV+ART- females, aged 30-49	102867	Social contact survey
contact_time_other_f_age1_HIV2	Minutes of contact time per month in other settings for HIV+ART+ females, aged 30-49	80278	Social contact survey
contact_time_other_f_age2_HIV01	Minutes of contact time per month in other settings for HIV- females and HIV+ART- females, aged 50+	98754	Social contact survey
contact_time_other_f_age2_HIV2	Minutes of contact time per month in other settings for HIV+ART+ females, aged 50+	76164	Social contact survey
contact_time_clinic_m_HIV0_low	Minutes of contact time per month in clinics for HIV- males, in the low clinic visiting group	493 (varied in sensitivity analyses)	Social contact survey
contact_time_clinic_m_HIV1_low	Minutes of contact time per month in clinics for HIV+ART- males, in the low clinic visiting group	493 (varied in sensitivity analyses)	Social contact survey
contact_time_clinic_m_HIV2_low	Minutes of contact time per month in clinics for HIV+ART+ males, in the low clinic visiting group	3468 (varied in sensitivity analyses)	Social contact survey
contact_time_clinic_f_HIV0_low	Minutes of contact time per month in clinics for HIV- females, in the low clinic visiting group	2322 (varied in sensitivity analyses)	Social contact survey
contact_time_clinic_f_HIV1_low	Minutes of contact time per month in clinics for HIV+ART- females, in the low clinic visiting group	2322 (varied in sensitivity analyses)	Social contact survey

contact_time_clinic_f_HIV2_low	Minutes of contact time per month in clinics for HIV+ART+ females, in the low clinic visiting group	8276 (varied in sensitivity analyses)	Social contact survey
contact_time_clinic_m_HIV0_high	Minutes of contact time per month in clinics for HIV- males, in the high clinic visiting group	5507 (varied in sensitivity analyses)	Social contact survey
contact_time_clinic_m_HIV1_high	Minutes of contact time per month in clinics for HIV+ART- males, in the high clinic visiting group	5507 (varied in sensitivity analyses)	Social contact survey
contact_time_clinic_m_HIV2_high	Minutes of contact time per month in clinics for HIV+ART+ males, in the high clinic visiting group	8400 (varied in sensitivity analyses)	Social contact survey
contact_time_clinic_f_HIV0_high	Minutes of contact time per month in clinics for HIV- females, in the high clinic visiting group	8609 (varied in sensitivity analyses)	Social contact survey
contact_time_clinic_f_HIV1_high	Minutes of contact time per month in clinics for HIV+ART- females, in the high clinic visiting group	8609 (varied in sensitivity analyses)	Social contact survey
contact_time_clinic_f_HIV2_high	Minutes of contact time per month in clinics for HIV+ART+ females, in the high clinic visiting group	8276 (varied in sensitivity analyses)	Social contact survey
increased_contact_time_clinics_tb	Increased contact time in clinics for people with TB compared to people without	>1	Varied freely, to fit data
clinic_rate_switch_prob	Probability of switching clinic visiting group every six months	0.25 (varied in sensitivity analysis)	Plausible value. Effects explored in sensitivity analysis
ventilation_weight_home	Modifier of *transmission_prob* for contact time between household members, incorporating effects of different mean ventilation rates by location type	2.8	Lygizos *et al* 2013[4] and Beckwith *et al*[5]

ventilation_weight_clinic	Modifier of *transmission_prob* for contact time in clinics, incorporating effects of different mean ventilation rates by location type	1 (varied in sensitivity analysis)	
ventilation_weight_other	Modifier of *transmission_prob* for contact time in other locations, incorporating effects of different mean ventilation rates by location type	1	Taylor *et al* 2016[6] and Beckwith *et al*[5]
**Intervention parameters**			
int_RR_trans_clinics	Modifier of risk of infection per minute contact occurring in clinics	1 until 2021, then value dependent on simulated intervention	See section 'Interventions'
int_RR_contact_clinics_HIV01	Modifier of mean contact hours in clinics for people who are HIV- or HIV+ART-	1 until 2021, then value dependent on simulated intervention	See section 'Interventions'
int_RR_contact_clinics_HIV2	Modifier of mean contact hours in clinics for people who are HIV+ART+	1 until 2021, then value dependent on simulated intervention	See section 'Interventions'

Table S9. Description of model input parameters, values or plausible ranges, and data sources.

2.16 Model fitting targets

Description	Calibration target/Plausible range	Source
Growth in population size between 2015 and 2019	3.4%	Mid-year population estimates 2019[43]
Proportion of the population who are male in 2018	48%	Mid-year population estimates 2018[44]
Proportion of simulated men aged 15-29	43%	Mid-year population estimates 2018[44]
Proportion of simulated men aged 30-49	39%	Mid-year population estimates 2018[44]
Proportion of simulated men aged 50+	18%	Mid-year population estimates 2018[44]
Proportion of simulated women aged 15-29	38%	Mid-year population estimates 2018[44]
Proportion of simulated women aged 30-49	36%	Mid-year population estimates 2018[44]
Proportion of simulated women aged 50+	25%	Mid-year population estimates 2018[44]
HIV prevalence in men aged 15-29, in 2011	7%	Vandormael *et al* (2019)[17]
HIV prevalence in men aged 30-49, in 2011	48%	Vandormael *et al* (2019)[17]
HIV prevalence in women aged 15-29, in 2011	26%	Vandormael *et al* (2019)[17]
HIV prevalence in women aged 30-49, in 2011	48%	Vandormael *et al* (2019)[17]
HIV prevalence in men aged 15-29, in 2017	8%	Vandormael *et al* (2019)[17]
HIV prevalence in men aged 30-49, in 2017	44%	Vandormael *et al* (2019)[17]
HIV prevalence in men aged 50+, in 2017	30%	Vandormael *et al* (2019)[17]
HIV prevalence in women aged 15-29, in 2017	25%	Vandormael *et al* (2019)[17]
HIV prevalence in women aged 30-49, in 2017	59%	Vandormael *et al* (2019)[17]

HIV prevalence in women aged 50+, in 2017	35%	Vandormael *et al* (2019)[17]
Proportion of HIV positive people on ART in 2012	25-45%	Vandormael *et al* (2019)[17]
Proportion of HIV positive people aged 15-29 on ART in 2017	49%	Vandormael *et al* (2019)[17]
Proportion of HIV positive people aged 30-49 on ART in 2017	74%	Vandormael *et al* (2019)[17]
Proportion of HIV positive people aged 50+ on ART in 2017	86%	Vandormael *et al* (2019)[17]
Proportion of HIV positive men on ART in 2017	63%	Vandormael *et al* (2019)[17]
Proportion of HIV positive women on ART in 2017	73%	Vandormael *et al* (2019)[17]
Annual incidence of TB per 100,000 population in 2011	1433 (1107-1803)	Notification data for KZN[28], adjusted for under-reporting, assuming that the proportion of cases notified is the same for KZN as for South Africa as a whole[11]
Annual incidence of TB per 100,000 population in 2018	658 (472-874)	Notification data for KZN[28], adjusted for under-reporting, assuming that the proportion of cases notified is the same for KZN as for South Africa as a whole[11]
Proportion of incident TB that is in HIV positive people in 2018	0.58	Data on patients starting TB treatment in KZN[28], assuming that the proportion of incident TB that is in HIV positive people is the same as the proportion of people starting TB treatment who are HIV positive (as is assumed by WHO for South Africa as a whole[11]).
Proportion of incident TB that is MDR in 2012	0.029	Ismail *et al* 2018[38]
Proportion of incident TB that is MDR in 2018	0.031	Estimated proportion of TB cases starting treatment in 2018 who have MDR TB. Unpublished, provisional data from the National Institute for Communicable Diseases

Annual HIV negative TB mortality rate per 100,000 population in 2018	47 (34-63)	Calculated from estimated incidence in HIV- people in KZN in 2018, and estimated case fatality ratio for TB in HIV- people in South Africa[11]
Annual HIV positive TB mortality rate per 100,000 population in 2018	92 (66-122)	Calculated from estimated incidence in HIV positive people in KZN in 2018, and estimated case fatality ratio for TB in HIV positive people in South Africa[11]
Proportion of people starting TB treatment who are HIV positive in 2018	0.58	Data on patients starting TB treatment in KZN[28]
Ratio of cases starting treatment to estimated incidence in 2000	57% (40%-89%)	WHO global TB report 2019[11]
Ratio of cases starting treatment to estimated incidence in 2018	76% (57%-100%)	WHO global TB report 2019[11]
Proportion starting treatment in 2017 who complete treatment, DS TB	78%	WHO global TB report 2019[11]
Proportion starting treatment in 2017 who complete treatment, MDR TB	54%	WHO global TB report 2019[11]
Proportion starting treatment in 2017 who die while on treatment, DS TB	11%	Data from KZN[28]
Proportion starting treatment in 2017 who die while on treatment, MDR TB	23%	Data from KZN[28]
Proportion starting treatment in 2017 who dropped out of treatment, DS TB	11%	Data from KZN[28]

Proportion starting treatment in 2017 who dropped out of treatment, MDR TB	23%	Data from KZN[28]
Increased prevalence of TB in clinic attendees, compared to the general population, in 2019	1.86	Govender *et al* (2020)[3]
Proportion of incident TB that results from transmission between household members, in 2018	13.5%	McCreesh and White (2018)[14]
Relative change in HIV prevalence in men between 2020 and 2030	-16.2%	Estimates from Thembisa model[18 19]
Relative change in HIV prevalence in women between 2020 and 2030	-5.7%	Estimates from Thembisa model[18 19]
Relative change in ART coverage among HIV+ men between 2020 and 2030	5.4%	Estimates from Thembisa model[18 19]
Relative change in ART coverage among HIV+ women between 2020 and 2030	2.0%	Estimates from Thembisa model[18 19]

Table S10. Model fitting targets in the best estimate scenario. Where no ranges are given, fits were considered acceptable if they were within ±20% of the target value.

3 Model results

3.1 Calibrated input parameter values

	Best estimate	Proportion of outside-household contact time occurring in clinics		Proportion of disease from household transmission		Ventilation rates in clinics		Prevalence of TB in clinic attendees		Movement between high and low clinic visiting groups		HIV+ART clinic visiting		Future HIV incidence	
		Low	High	Low	High	Low	High	Low	High	Low	High	Low	High	Low	High
birth_rate	0.0021	0.0021	0.0021	0.0021	0.0021	0.0021	0.0021	0.0021	0.0021	0.0021	0.0021	0.0021	0.0021	0.0021	0.0021
mortality_rate_m_age0	0	0	0	0	0	0	0	0	0	0	0	0	0	0	0
mortality_rate_m_age1	0	0	0	0	0	0	0	0	0	0	0	0	0	0	0
mortality_rate_m_age2	0.055	0.055	0.055	0.055	0.055	0.055	0.055	0.055	0.055	0.055	0.055	0.055	0.055	0.055	0.055
mortality_rate_f_age0	0	0	0	0	0	0	0	0	0	0	0	0	0	0	0
mortality_rate_f_age1	0	0	0	0	0	0	0	0	0	0	0	0	0	0	0
mortality_rate_f_age2	0.025	0.025	0.025	0.025	0.025	0.025	0.025	0.025	0.025	0.025	0.025	0.025	0.025	0.025	0.025
transmission_prob_early	1.11E-05	1.16E-05	1.09E-05	1.03E-05	1.18E-05	1.13E-05	1.04E-05	1.13E-05	1.06E-05	1.11E-05	1.11E-05	1.11E-05	1.11E-05	1.11E-05	1.10E-05
TB_parameter_change_year	2014	2014	2014	2014	2014	2014	2014	2014	2014	2014	2014	2014	2014	2014	2014
decreased_tb_rates_late	0.86	0.86	0.86	0.86	0.86	0.86	0.86	0.86	0.86	0.86	0.86	0.86	0.86	0.86	0.86
treatment_rate_HIV0_early	0.48	0.48	0.48	0.48	0.48	0.48	0.48	0.48	0.48	0.48	0.48	0.48	0.48	0.48	0.48
treatment_rate_HIV12_early	0.78	0.78	0.78	0.78	0.78	0.78	0.78	0.78	0.78	0.78	0.78	0.78	0.78	0.78	0.78
treatment_rate_HIV0_late	0.68	0.68	0.68	0.68	0.68	0.68	0.68	0.68	0.68	0.68	0.68	0.68	0.68	0.68	0.68
treatment_rate_HIV12_late	0.81	0.81	0.81	0.81	0.81	0.81	0.81	0.81	0.81	0.81	0.81	0.81	0.81	0.81	0.81
treatment_rate_HIV0_late	0.68	0.68	0.68	0.68	0.68	0.68	0.68	0.68	0.68	0.68	0.68	0.68	0.68	0.68	0.68
treatment_rate_HIV12_late	0.81	0.81	0.81	0.81	0.81	0.81	0.81	0.81	0.81	0.81	0.81	0.81	0.81	0.81	0.81
TB_mortality_rate_smearneg_HIV0	0.025	0.025	0.025	0.025	0.025	0.025	0.025	0.025	0.025	0.025	0.025	0.025	0.025	0.025	0.025
TB_mortality_rate_smearneg_HIV1	0.60	0.60	0.60	0.60	0.60	0.60	0.60	0.60	0.60	0.60	0.60	0.60	0.60	0.60	0.60
TB_mortality_rate_smearneg_HIV2	0.10	0.10	0.10	0.10	0.10	0.10	0.10	0.10	0.10	0.10	0.10	0.10	0.10	0.10	0.10
TB_mortality_rate_smearpos_HIV0	0.39	0.39	0.39	0.39	0.39	0.39	0.39	0.39	0.39	0.39	0.39	0.39	0.39	0.39	0.39

TB_mortality_rate_smearpos_HIV1	0.80	0.80	0.80	0.80	0.80	0.80	0.80	0.80	0.80	0.80	0.80	0.80	0.80	0.80	0.80
TB_mortality_rate_smearpos_HIV2	0.39	0.39	0.39	0.39	0.39	0.39	0.39	0.39	0.39	0.39	0.39	0.39	0.39	0.39	0.39
TB_mortality_rate_treatment_DS	0.12	0.12	0.12	0.12	0.12	0.12	0.12	0.12	0.12	0.12	0.12	0.12	0.12	0.12	0.12
TB_mortality_rate_treatment_MDR	0.14	0.14	0.14	0.14	0.14	0.14	0.14	0.14	0.14	0.14	0.14	0.14	0.14	0.14	0.14
TB_treatment_dropout_rate_DS	0.011	0.011	0.011	0.011	0.011	0.011	0.011	0.011	0.011	0.011	0.011	0.011	0.011	0.011	0.011
TB_treatment_dropout_rate_MDR	0.011	0.011	0.011	0.011	0.011	0.011	0.011	0.011	0.011	0.011	0.011	0.011	0.011	0.011	0.011
develop_tb_reactivation_rate_HIV1_early	0.20	0.20	0.20	0.20	0.20	0.20	0.20	0.20	0.20	0.20	0.20	0.20	0.20	0.20	0.20
develop_tb_reactivation_rate_HIV1_late	0.080	0.080	0.080	0.080	0.080	0.080	0.080	0.080	0.080	0.080	0.080	0.080	0.080	0.080	0.080
reinfection_relative_risk_HIV1_early	0.90	0.90	0.90	0.90	0.90	0.90	0.90	0.90	0.90	0.90	0.90	0.90	0.90	0.90	0.90
reinfection_relative_risk_HIV1_late	0.60	0.60	0.60	0.60	0.60	0.60	0.60	0.60	0.60	0.60	0.60	0.60	0.60	0.60	0.60
reinfection_relative_risk_HIV2	0.50	0.50	0.50	0.50	0.50	0.50	0.50	0.50	0.50	0.50	0.50	0.50	0.50	0.50	0.50
change_HIV0_parameters_year	2007	2007	2007	2007	2007	2007	2007	2007	2007	2007	2007	2007	2007	2007	2007
hiv_inc_early_f0	0.066	0.066	0.066	0.066	0.066	0.066	0.066	0.066	0.066	0.066	0.066	0.066	0.066	0.066	0.066
hiv_inc_early_f1	0.081	0.081	0.081	0.081	0.081	0.081	0.081	0.081	0.081	0.081	0.081	0.081	0.081	0.081	0.081
hiv_inc_early_f2	0.023	0.023	0.023	0.023	0.023	0.023	0.023	0.023	0.023	0.023	0.023	0.023	0.023	0.023	0.023
hiv_inc_early_m0	0.015	0.015	0.015	0.015	0.015	0.015	0.015	0.015	0.015	0.015	0.015	0.015	0.015	0.015	0.015
hiv_inc_early_m1	0.12	0.12	0.12	0.12	0.12	0.12	0.12	0.12	0.12	0.12	0.12	0.12	0.12	0.12	0.12
hiv_inc_early_m2	0	0	0	0	0	0	0	0	0	0	0	0	0	0	0
hiv_inc_mid_f0	0.055	0.055	0.055	0.055	0.055	0.055	0.055	0.055	0.055	0.055	0.055	0.055	0.055	0.055	0.055
hiv_inc_mid_f1	0.06	0.06	0.06	0.06	0.06	0.06	0.06	0.06	0.06	0.06	0.06	0.06	0.06	0.06	0.06
hiv_inc_mid_f2	0.023	0.023	0.023	0.023	0.023	0.023	0.023	0.023	0.023	0.023	0.023	0.023	0.023	0.023	0.023
hiv_inc_mid_m0	0.017	0.017	0.017	0.017	0.017	0.017	0.017	0.017	0.017	0.017	0.017	0.017	0.017	0.017	0.017
hiv_inc_mid_m1	0.071	0.071	0.071	0.071	0.071	0.071	0.071	0.071	0.071	0.071	0.071	0.071	0.071	0.071	0.071
hiv_inc_mid_m2	0	0	0	0	0	0	0	0	0	0	0	0	0	0	0
HIV_inc_reduction_late_m	0.46	0.46	0.46	0.46	0.46	0.46	0.46	0.46	0.46	0.46	0.46	0.46	0.46	0.23	0.73
HIV_inc_reduction_late_f	0.32	0.32	0.32	0.32	0.32	0.32	0.32	0.32	0.32	0.32	0.32	0.32	0.32	0.16	0.66
ART_start_rate_early_m	0.032	0.032	0.032	0.032	0.032	0.032	0.032	0.032	0.032	0.032	0.032	0.032	0.032	0.032	0.032
ART_start_rate_early_f	0.048	0.048	0.048	0.048	0.048	0.048	0.048	0.048	0.048	0.048	0.048	0.048	0.048	0.048	0.048

McCreesh N, *et al. BMJ Global Health* 2022; 7:e007136. doi: 10.1136/bmjgh-2021-007136

ART_start_rate_late_m	0.16	0.16	0.16	0.16	0.16	0.16	0.16	0.16	0.16	0.16	0.16	0.16	0.16	0.16	0.16
ART_start_rate_late_f	0.22	0.22	0.22	0.22	0.22	0.22	0.22	0.22	0.22	0.22	0.22	0.22	0.22	0.22	0.22
increased_contact_time_clinics_tb	1.42	1.42	1.42	1.42	1.42	1.42	1.42	1.00	2.42	1.42	1.42	1.42	1.42	1.42	1.42
infectiousness_var	33	35	33	18	72	33	33	33	33	33	33	33	33	33	33

Table S11. Fitted input parameter values in the best estimate scenario and sensitivity analysis scenarios. 'Low' and 'high' refer to changes that decrease and increase the proportion of disease that results from transmission in clinics respectively. Parameter names are given in bold if the fitted value varied between scenarios

3.2 Fit to data

	Target (best estimate scenario)	Best estimate	Proportion of outside-household contact time occurring in clinics		Proportion of disease from household transmission		Ventilation rates in clinics		Prevalence of TB in clinic attendees		Movement between high and low clinic visiting groups		HIV+ART- clinic visiting		Future HIV incidence	
			Low	High	Low	High	Low	High	Low	High	Low	High	Low	High	Low	High
Growth in population size between 2015 and 2019	0.034	0.033	0.033	0.034	0.033	0.033	0.033	0.033	0.033	0.033	0.033	0.033	0.033	0.034	0.033	0.033
Proportion of the population who are male in 2018	0.48	0.50	0.50	0.50	0.50	0.50	0.50	0.50	0.50	0.50	0.50	0.50	0.50	0.50	0.50	0.50
Proportion of simulated men aged 15-29	0.43	0.46	0.46	0.46	0.46	0.46	0.46	0.46	0.46	0.46	0.46	0.46	0.46	0.46	0.46	0.46
Proportion of simulated men aged 30-49	0.39	0.39	0.39	0.39	0.39	0.39	0.39	0.39	0.39	0.39	0.39	0.39	0.39	0.39	0.39	0.39
Proportion of simulated men aged 50+	0.18	0.15	0.15	0.15	0.15	0.15	0.15	0.15	0.15	0.15	0.15	0.15	0.15	0.15	0.15	0.15
Proportion of simulated women aged 15-29	0.38	0.43	0.43	0.43	0.43	0.43	0.43	0.43	0.43	0.43	0.43	0.43	0.43	0.43	0.43	0.43
Proportion of simulated women aged 30-49	0.36	0.33	0.33	0.33	0.33	0.33	0.33	0.33	0.33	0.33	0.33	0.33	0.33	0.33	0.33	0.33
Proportion of simulated women aged 50+	0.25	0.24	0.24	0.24	0.24	0.24	0.24	0.24	0.24	0.24	0.24	0.24	0.24	0.24	0.24	0.24
HIV prevalence in men aged 15-29, in 2011	0.070	0.074	0.073	0.073	0.07	0.073	0.074	0.074	0.073	0.074	0.074	0.074	0.074	0.073	0.074	0.074
HIV prevalence in men aged 30-49, in 2011	0.48	0.50	0.50	0.50	0.50	0.50	0.50	0.50	0.50	0.50	0.50	0.50	0.50	0.50	0.50	0.50
HIV prevalence in women aged 15-29, in 2011	0.26	0.27	0.27	0.27	0.27	0.27	0.27	0.27	0.27	0.27	0.27	0.27	0.27	0.27	0.27	0.27

HIV prevalence in women aged 30-49, in 2011	0.48	0.50	0.50	0.49	0.50	0.50	0.50	0.50	0.50	0.50	0.50	0.50	0.50	0.49	0.50	0.50
HIV prevalence in men aged 15-29, in 2017	0.080	0.083	0.083	0.083	0.08	0.083	0.083	0.083	0.083	0.083	0.083	0.083	0.083	0.083	0.083	0.083
HIV prevalence in men aged 30-49, in 2017	0.44	0.45	0.45	0.45	0.45	0.45	0.45	0.45	0.45	0.45	0.45	0.45	0.45	0.45	0.45	0.45
HIV prevalence in men aged 50+, in 2017	0.30	0.34	0.34	0.34	0.34	0.34	0.34	0.34	0.34	0.34	0.34	0.34	0.34	0.34	0.34	0.34
HIV prevalence in women aged 15-29, in 2017	0.25	0.26	0.26	0.26	0.26	0.26	0.26	0.26	0.26	0.26	0.26	0.26	0.26	0.26	0.26	0.26
HIV prevalence in women aged 30-49, in 2017	0.59	0.56	0.56	0.56	0.57	0.56	0.56	0.56	0.56	0.56	0.56	0.56	0.57	0.56	0.56	0.57
HIV prevalence in women aged 50+, in 2017	0.35	0.39	0.39	0.39	0.39	0.39	0.39	0.39	0.39	0.39	0.39	0.39	0.39	0.39	0.39	0.39
Proportion of HIV positive people on ART in 2012	25-45%	0.32	0.32	0.32	0.32	0.32	0.32	0.32	0.32	0.32	0.32	0.32	0.32	0.32	0.32	0.32
Proportion of HIV positive people aged 15-29 on ART in 2017	0.49	0.57	0.57	0.57	0.57	0.57	0.57	0.57	0.57	0.57	0.57	0.57	0.57	0.57	0.57	0.57
Proportion of HIV positive people aged 30-49 on ART in 2017	0.74	0.70	0.70	0.70	0.70	0.70	0.70	0.70	0.70	0.70	0.70	0.70	0.70	0.70	0.70	0.70
Proportion of HIV positive people aged 50+ on ART in 2017	0.86	0.79	0.79	0.79	0.79	0.79	0.79	0.79	0.79	0.79	0.79	0.79	0.79	0.80	0.79	0.79
Proportion of HIV positive men on ART in 2017	0.63	0.63	0.63	0.63	0.63	0.63	0.63	0.63	0.63	0.63	0.63	0.63	0.63	0.63	0.63	0.63
Proportion of HIV positive women on ART in 2017	0.73	0.73	0.73	0.73	0.73	0.73	0.73	0.73	0.73	0.73	0.73	0.73	0.73	0.73	0.73	0.73
Annual incidence of TB per 100,000 population in 2011	1433 (1107-1803)	1194	1240	1228	1157	1193	1150	1175	1201	1179	1198	1242	1156	1256	1196	1161
Annual incidence of TB per 100,000 population in 2018	658 (472-874)	631	660	636	616	626	600	635	628	630	632	660	612	655	630	614
Proportion of incident TB that is in HIV positive people in 2018	0.58	0.55	0.55	0.55	0.55	0.55	0.54	0.56	0.55	0.55	0.55	0.55	0.54	0.55	0.55	0.55
Proportion of incident TB that is MDR in 2012	0.029	0.027	0.028	0.027	0.03	0.027	0.027	0.027	0.027	0.027	0.027	0.027	0.027	0.027	0.027	0.027
Proportion of incident TB that is MDR in 2018	0.031	0.033	0.034	0.034	0.03	0.033	0.033	0.033	0.034	0.034	0.033	0.033	0.033	0.033	0.033	0.033
Annual HIV- TB mortality rate per 100,000 population in 2018	47 (34-63)	57	59	58	56	56	55	57	57	56	57	59	56	58	57	56

McCreesh N, *et al. BMJ Global Health* 2022; 7:e007136. doi: 10.1136/bmjgh-2021-007136

Annual HIV positive TB mortality rate per 100,000 population in 2016	92 (66-122)	101	106	102	97	100	96	102	100	101	101	106	97	107	100	98
Proportion of people starting TB treatment who are HIV positive in 2018	0.58	0.55	0.55	0.54	0.54	0.55	0.54	0.55	0.54	0.55	0.55	0.55	0.54	0.55	0.55	0.54
Ratio of cases starting treatment to estimated incidence in 2000	57% (40-89)	0.49	0.49	0.48	0.49	0.49	0.49	0.49	0.49	0.49	0.49	0.49	0.49	0.48	0.49	0.49
Ratio of cases starting treatment to estimated incidence in 2018	76% (57-110)	0.78	0.78	0.78	0.78	0.78	0.78	0.78	0.78	0.78	0.78	0.78	0.78	0.78	0.78	0.78
Proportion starting treatment in 2017 who complete treatment, DS TB	0.78	0.78	0.78	0.78	0.79	0.79	0.78	0.78	0.78	0.78	0.78	0.78	0.79	0.78	0.79	0.78
Proportion starting treatment in 2017 who complete treatment, MDR TB	0.54	0.54	0.54	0.54	0.54	0.55	0.53	0.52	0.54	0.54	0.53	0.54	0.53	0.54	0.54	0.54
Proportion starting treatment in 2017 who die while on treatment, DS TB	0.11	0.11	0.11	0.11	0.11	0.11	0.11	0.11	0.11	0.11	0.11	0.11	0.11	0.11	0.11	0.11
Proportion starting treatment in 2017 who die while on treatment, MDR TB	0.23	0.22	0.21	0.21	0.22	0.21	0.22	0.22	0.21	0.22	0.22	0.22	0.21	0.21	0.21	0.22
Proportion starting treatment in 2017 who dropped out of treatment, DS TB	0.11	0.11	0.11	0.11	0.10	0.10	0.11	0.11	0.11	0.11	0.10	0.11	0.10	0.11	0.10	0.10
Proportion starting treatment in 2017 who dropped out of treatment, MDR TB	0.23	0.23	0.24	0.23	0.22	0.22	0.23	0.24	0.23	0.24	0.24	0.23	0.24	0.24	0.24	0.23
Increased prevalence of TB in clinic attendees, compared to the general population	1.86	1.83	1.83	1.83	1.83	1.83	1.82	1.83	1.29*	3.09*	1.84	1.83	1.84	1.81	1.83	1.83
Proportion of incident TB that results from transmission between household members, in 2018	8-19%	0.12	0.12	0.12	0.17*	0.07*	0.12	0.12	0.12	0.12	0.12	0.12	0.12	0.12	0.12	0.12
Relative change in HIV prevalence in men between 2020 and 2030‡	-0.16	-0.16	-0.16	-0.16	-0.16	-0.16	-0.16	-0.16	-0.16	-0.16	-0.16	-0.16	-0.16	-0.16	-0.26*	0.038*
Relative change in HIV prevalence in women between 2020 and 2030‡	-0.057	-0.058	-0.058	-0.058	-0.059	-0.058	-0.058	-0.059	-0.058	-0.059	-0.058	-0.058	-0.058	-0.058	-0.17*	0.067*

Table S12. Model fit to fitting targets, in the best estimate scenario and sensitivity analysis scenarios. ‘Low’ and ‘high’ refer to changes that decrease and increase the proportion of disease that results from transmission in clinics respectively. *Indicates fitting outputs where the target value was changed in the sensitivity analysis. ┼Indicates outputs where the value could change in the intervention scenarios. Figures shown are for the baseline scenario

3.3 Results by uncertainty analysis scenario

3.3.1 Proportion of disease from transmission in clinics

Figure S3 shows the proportion of disease that resulted from transmission in clinics in the study population in 2019, by scenario and by population group. The sources of uncertainty in model input parameters that had the largest effect on model estimates were the amount of contact time that occurred in clinics, the prevalence of TB in clinic attendees compared to the general population, and ventilation levels in clinics relative to in other settings. The proportion of disease that results from transmission in households, and the rate at which individuals switched between high and low clinic visiting groups, had little effect on model estimates.

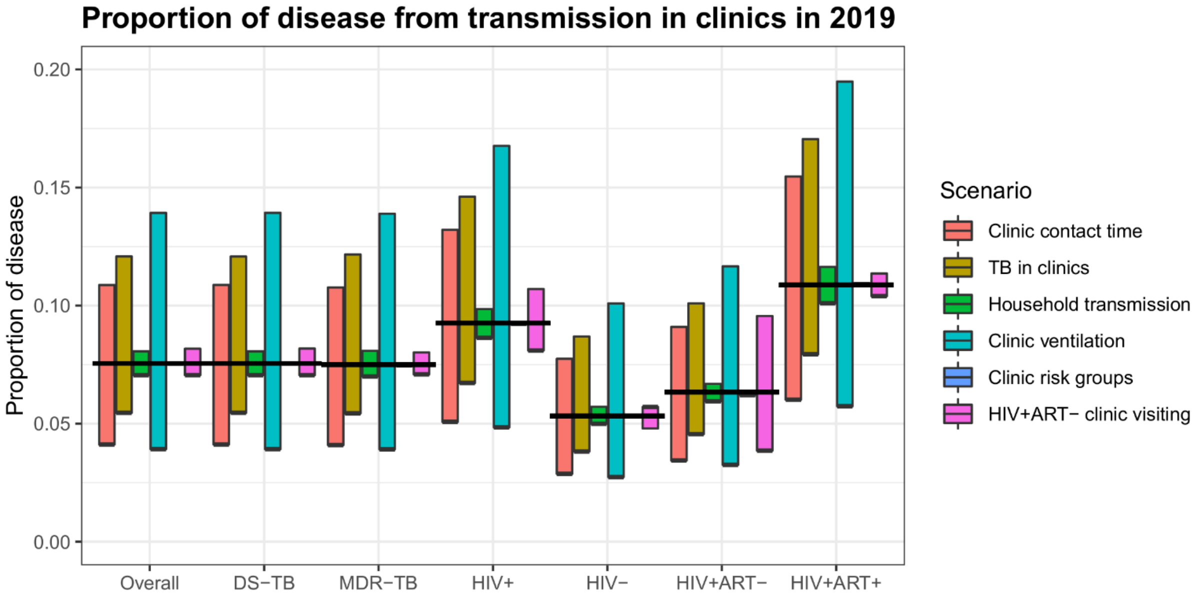

Figure S3. The estimated proportion of disease that resulted from transmission in clinics in the study population in 2019, by scenario and by population group. Horizontal black lines show the estimates from the 'best estimate' scenario. See section 'Uncertainty analyses' for a description of the scenarios. The 'Clinic risk groups' uncertainty analysis had little effect on the results, and therefore the bar is mostly hidden under the horizontal black lines.

3.3.2 Intervention impact

Figures S4-S7 show the estimated reductions in TB cases and TB deaths, overall and MDR-TB, in the study population in 2021-2030, by intervention and scenario.

McCreesh N, *et al. BMJ Global Health* 2022; 7:e007136. doi: 10.1136/bmjgh-2021-007136

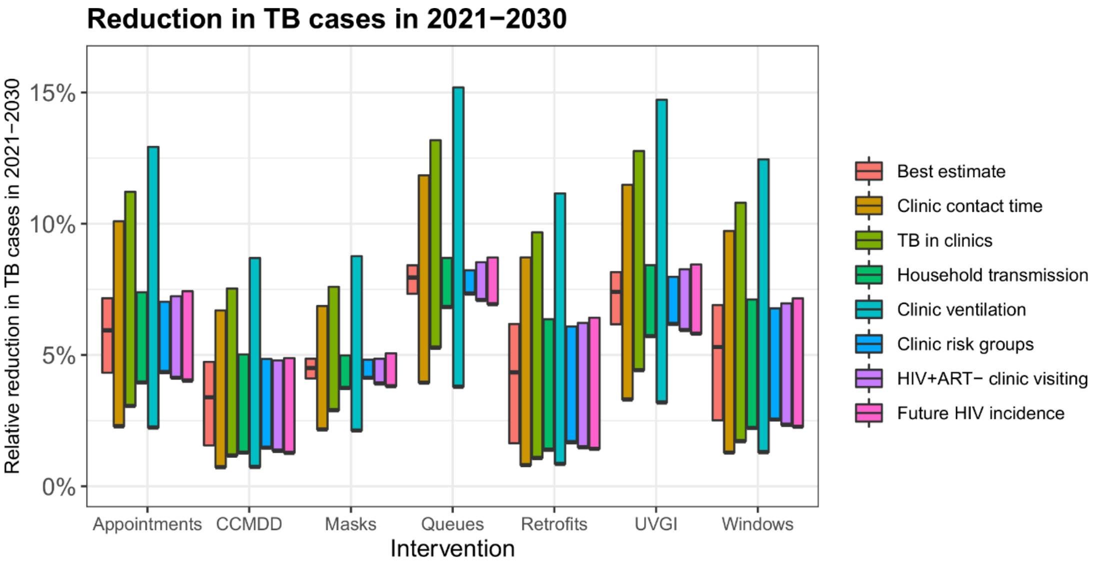

Figure S4. The estimated reduction in TB cases in the study population in 2021-2030 resulting from the proposed infection prevention and control interventions, by scenario.

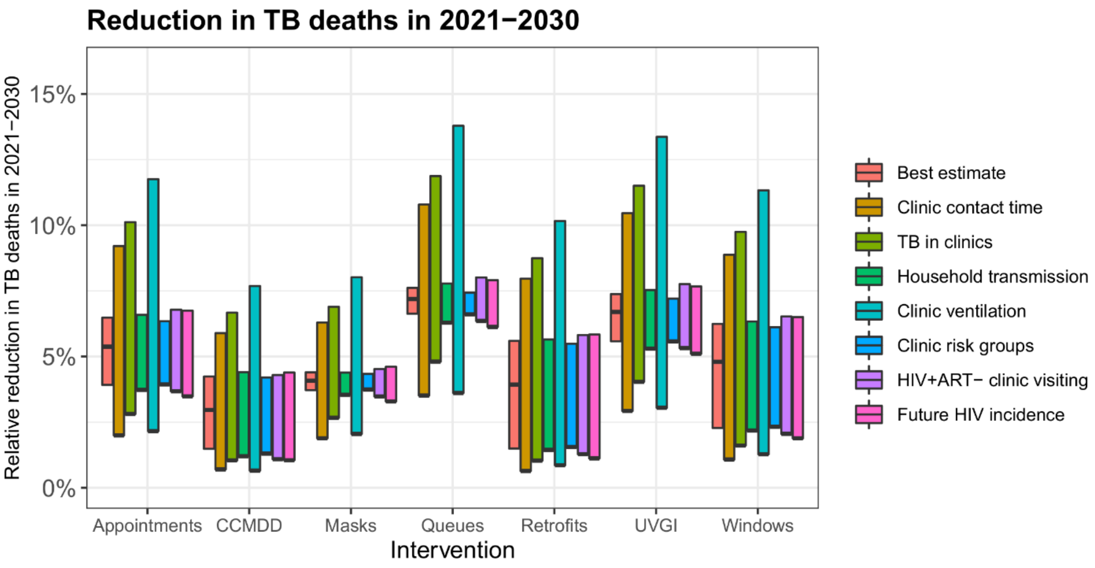

Figure S5. The estimated reduction in TB deaths in the study population in 2021-2030 resulting from the proposed infection prevention and control interventions, by scenario.

McCreesh N, *et al. BMJ Global Health* 2022; 7:e007136. doi: 10.1136/bmjgh-2021-007136

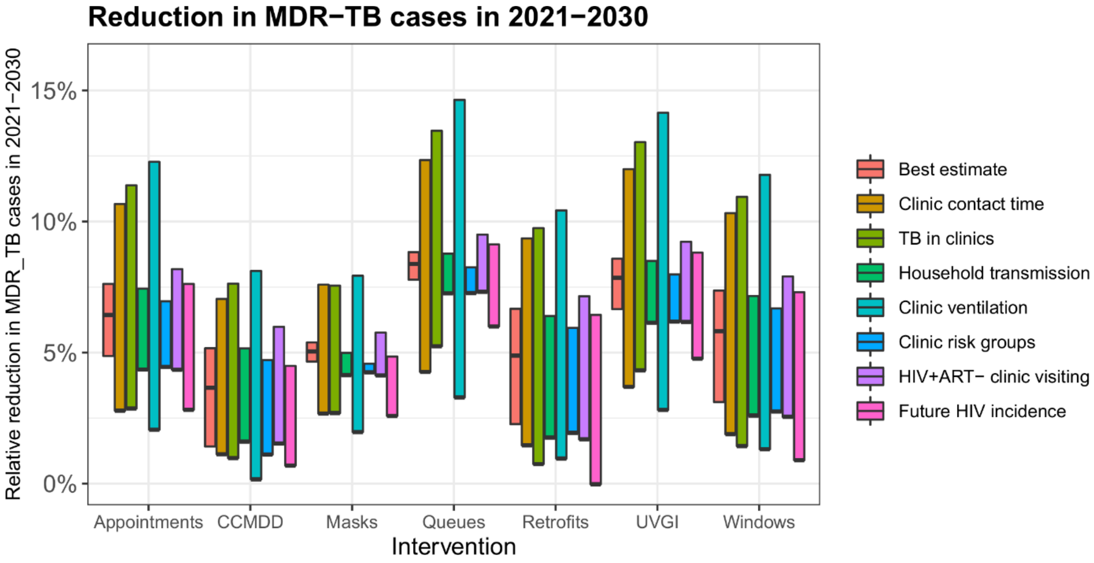

Figure S6. The estimated reduction in MDR-TB cases in the study population in 2021-2030 resulting from the proposed infection control interventions, by scenario.

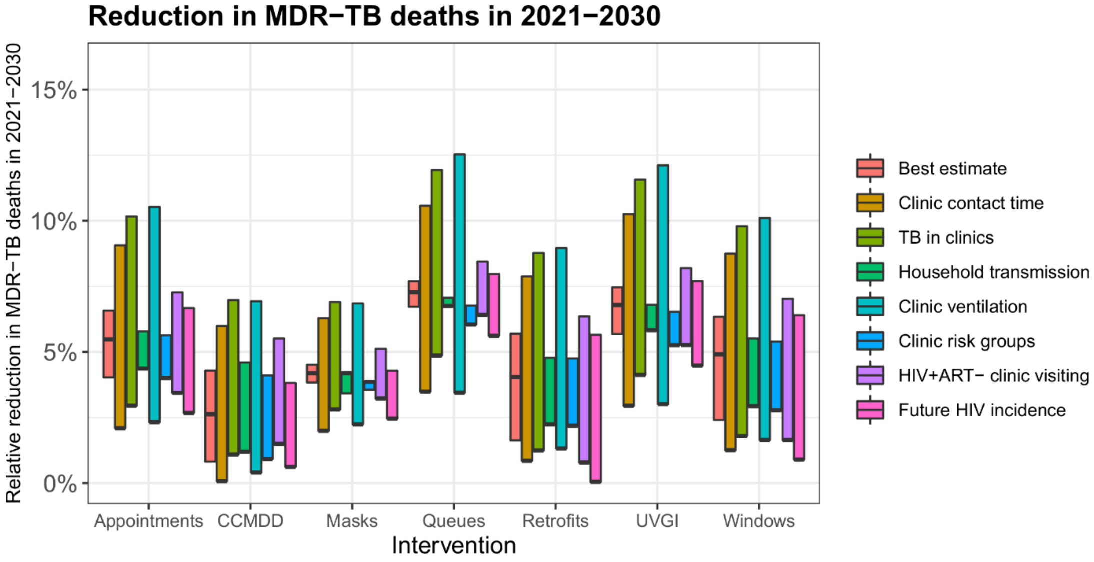

Figure S7. The estimated reduction in MDR-TB deaths in the study population in 2021-2030 resulting from the proposed infection control interventions, by scenario.

4 Proportion of disease from transmission in clinics that is in clinic staff

4.1 Methods

In the mathematical model, we only consider transmission to adult clinic attendees (patients, and people attending with or on the behalf of patients). Clinic staff are also at risk of infection in clinics however, and here we use a simple calculation to obtain a rough estimate of the proportion of tuberculosis in adults resulting from transmission in clinics that is in clinic staff.

The proportion can be estimated using the following equation:

$$p = s(s + c(1 - s)(r - 1)^{-1})^{-1}$$

Where:

- *p* is the proportion of all disease resulting from transmission in clinics that is in clinic staff
- *s* is the proportion of the population who are clinic staff
- *c* is the proportion of disease that results from transmission in clinics in the general population
- *r* is the relative rate of TB in clinic staff compared to the general population

Assuming that all clinic staff who are at elevated risk of infection from transmission in clinics have the same exposure to TB outside the clinic as the general population, and that all excess TB in clinic staff results from transmission in clinics.

Two clinics serve the study population. The clinics have a total of 59 staff who are considered to be at elevated risk of infection from transmission in clinics, with the rest being situated outside the majority of the time (e.g. security guards), or spending little time in public areas when patients are present. The adult total population of the study communities was 33,288. This means that *s = 59 / 33288.*

The results of this work indicate that 7.1% of disease in adults in the general population results from transmission in the clinic, with a plausible range of 4.0-14.2%.

No data were available on excess tuberculosis risk in clinic staff in our study setting. A recent systematic review of TB incidence in healthcare workers estimated that the ratio of the rate of TB in healthcare workers compared to the general population in high TB burden settings was 4.32 (95% CI 2.36-7.91[22]).

To generating a best estimate for *p*, we took the best estimates for all three parameters. To generate a 95% range, we generated 10,000 bootstrap samples, sampling *c* from uniform(0.04,0.142), and *r* from a split normal distribution with mean 4.32 and 95% CI 2.36-7.91. The 95% range was calculated as the 0.025th and 0.975th percentiles.

4.2 Results

We estimate that in the study community, an average of 7.1% (95% plausible range 2.3-16.7%) of all disease in adults resulting from transmission in clinics occurs in clinic staff.

5 References

1. McCreesh N, Dlamini V, Edwards A, et al. Impact of the Covid-19 epidemic and related social distancing regulations on social contact and SARS-CoV-2 transmission potential in rural South Africa: analysis of repeated cross-sectional surveys. *BMC infectious diseases* 2021;21(1):1-11.
2. Middelkoop K, Bekker L-G, Morrow C, et al. Decreasing household contribution to TB transmission with age: a retrospective geographic analysis of young people in a South African township. *BMC Infectious Diseases* 2014;14(1):221. doi: 10.1186/1471-2334-14-221
3. Govender I, Karat AS, Olivier S, et al. Prevalence of Mycobacterium tuberculosis in sputum and reported symptoms among clinic attendees compared to a community survey in rural South Africa. *Clinical Infectious Diseases* 2021
4. Lygizos M, Shenoi SV, Brooks RP, et al. Natural ventilation reduces high TB transmission risk in traditional homes in rural KwaZulu-Natal, South Africa. *BMC Infectious Diseases* 2013;13(1):300. doi: 10.1186/1471-2334-13-300
5. Beckwith P, Deol A, McCreesh N, et al. Direct estimates of absolute ventilation in primary health care clinics in South Africa.
6. Taylor JG, Yates TA, Mthethwa M, et al. Measuring ventilation and modelling M. tuberculosis transmission in indoor congregate settings, rural KwaZulu-Natal. *Int J Tuberc Lung Dis* 2016;20(9):1155-61. doi: 10.5588/ijtld.16.0085 [published Online First: 2016/08/12]
7. Organization WH. Rapid communication: key changes to treatment of multidrug-and rifampicin-resistant tuberculosis (MDR/RR-TB): World Health Organization, 2018.
8. Aurum Institute. Managing TB - In A New Era Of Diagnostics, 2016.
9. Glynn JR, Murray J, Bester A, et al. High rates of recurrence in HIV-infected and HIV-uninfected patients with tuberculosis. *The Journal of infectious diseases* 2010;201(5):704-11.
10. Verver S, Warren RM, Beyers N, et al. Rate of Reinfection Tuberculosis after Successful Treatment Is Higher than Rate of New Tuberculosis. *American Journal of Respiratory and Critical Care Medicine* 2005;171(12):1430-35. doi: 10.1164/rccm.200409-1200OC
11. World Health Organization. Global tuberculosis report 2019. Geneva, Switzerland: World Health Organization; 2019, 2019.
12. Yates TA. Mycobacterium tuberculosis infection in Southern Africa–exploring patterns, locating transmission. UCL (University College London), 2016.
13. Middelkoop K, Bekker L-G, Myer L, et al. Rates of tuberculosis transmission to children and adolescents in a community with a high prevalence of HIV infection among adults. 2008;47(3):349-55.

14. McCreesh N, White RG. An explanation for the low proportion of tuberculosis that results from transmission between household and known social contacts. *Scientific reports* 2018;8(1):5382.
15. Houben RMGJ, Lalli M, Sumner T, et al. TIME Impact – a new user-friendly tuberculosis (TB) model to inform TB policy decisions. *BMC Medicine* 2016;14(1):56. doi: 10.1186/s12916-016-0608-4
16. Dowdy DW, Chaisson RE. The persistence of tuberculosis in the age of DOTS: reassessing the effect of case detection. *Bulletin of the World Health Organization* 2009;87(4):296-304. doi: 10.2471/BLT.08.054510
17. Vandormael A, Akullian A, Siedner M, et al. Declines in HIV incidence among men and women in a South African population-based cohort. 2019;10(1):1-10.
18. Johnson LF, Dorrington RE, Moolla H. Progress towards the 2020 targets for HIV diagnosis and antiretroviral treatment in South Africa. *Southern African journal of HIV medicine* 2017;18(1)
19. https://thembisa.org/ [accessed 24/8/2020.
20. Diaconu K, Parkhurst J. Health systems webinar: Applying a 'whole systems' approach to infection prevention & control in primary health care clinics in South Africa. Using System Dynamics Modelling in Umoya omuhle 2021 [Available from: https://www.lshtm.ac.uk/research/centres-projects-groups/uo#events.
21. McCreesh N, Karat AS, Baisley K, et al. Modelling the effect of infection prevention and control measures on rate of Mycobacterium tuberculosis transmission to clinic attendees in primary health clinics in South Africa. *BMJ global health* 2021;6(10):e007124.
22. World Health Organization. WHO guidelines on tuberculosis infection prevention and control: 2019 update: World Health Organization 2019.
23. Chartier Y, Pessoa-Silva C. Natural ventilation for infection control in health-care settings: World Health Organization 2009.
24. Mphaphlele M, Dharmadhikari AS, Jensen PA, et al. Institutional tuberculosis transmission. Controlled trial of upper room ultraviolet air disinfection: A basis for new dosing guidelines. *American Journal of Respiratory and Critical Care Medicine* 2015;192(4):477-84. doi: 10.1164/rccm.201501-0060OC
25. Dharmadhikari AS, Mphahlele M, Stoltz A, et al. Surgical face masks worn by patients with multidrug-resistant tuberculosis: impact on infectivity of air on a hospital ward. 2012;185(10):1104-09.
26. MacIntyre CR, Chughtai AAJB. Facemasks for the prevention of infection in healthcare and community settings. 2015;350:h694.
27. Health Systems Trust. The CCMDD story, 2019.
28. HST Indicator Tool [Available from: https://indicators.hst.org.za/ accessed 7/4/2020 2020.
29. Karat AS, McCreesh N, Baisley K, et al. Waiting times, occupancy density, and patient flow in South African primary health clinics: implications for infection prevention and control. *MedRxiv* 2021;2021.07.21.21260806 doi: https://doi.org/10.1101/2021.07.21.21260806
30. Escombe AR, Ticona E, Chávez-Pérez V, et al. Improving natural ventilation in hospital waiting and consulting rooms to reduce nosocomial tuberculosis transmission risk in a low resource setting. *BMC infectious diseases* 2019;19(1):88.
31. Menzies NA, Cohen T, Lin H-H, et al. Population health impact and cost-effectiveness of tuberculosis diagnosis with Xpert MTB/RIF: a dynamic simulation and economic evaluation. *PloS Med* 2012;9(11):e1001347.
32. Ragonnet R, Flegg JA, Brilleman SL, et al. Revisiting the Natural History of Pulmonary Tuberculosis: A Bayesian Estimation of Natural Recovery and Mortality Rates. *Clinical Infectious Diseases* 2020 doi: 10.1093/cid/ciaa602
33. Dheda K, Lampe FC, Johnson MA, et al. Outcome of HIV-Associated Tuberculosis in the Era of Highly Active Antiretroviral Therapy. *The Journal of Infectious Diseases* 2004;190(9):1670-76. doi: 10.1086/424676

34. Lawn SD, Kranzer K, Wood RJCicm. Antiretroviral therapy for control of the HIV-associated tuberculosis epidemic in resource-limited settings. 2009;30(4):685-99.
35. Kasaie P, Andrews JR, Kelton WD, et al. Timing of Tuberculosis Transmission and the Impact of Household Contact Tracing: An Agent-Based Simulation Model. *American Journal of Respiratory and Critical Care Medicine* 2014;189(7):845-52. doi: 10.1164/rccm.201310-1846OC
36. Lawn SD, Myer L, Edwards D, et al. Short-term and long-term risk of tuberculosis associated with CD4 cell recovery during antiretroviral therapy in South Africa. 2009;23(13):1717.
37. Corbett EL, Watt CJ, Walker N, et al. The growing burden of tuberculosis: global trends and interactions with the HIV epidemic. *Archives of Internal Medicine* 2003;163(9):1009-21.
38. Ismail NA, Mvusi L, Nanoo A, et al. Prevalence of drug-resistant tuberculosis and imputed burden in South Africa: a national and sub-national cross-sectional survey. 2018;18(7):779-87.
39. Mandela N. Nelson Mandela/HSRC study of HIV/AIDS: South African national HIV prevalence, behavioural risks and mass media: household survey 2002: HSRC Press 2002.
40. Mossong J, Grapsa E, Tanser F, et al. Modelling HIV incidence and survival from age-specific seroprevalence after antiretroviral treatment scale-up in rural South Africa. *AIDS (London, England)* 2013;27(15):2471-79. doi: 10.1097/01.aids.0000432475.14992.da
41. Brinkhof MW, Boulle A, Weigel R, et al. Mortality of HIV-infected patients starting antiretroviral therapy in sub-Saharan Africa: comparison with HIV-unrelated mortality. 2009;6(4)
42. Granich R, Gupta S, Hersh B, et al. Trends in AIDS Deaths, New Infections and ART Coverage in the Top 30 Countries with the Highest AIDS Mortality Burden; 1990-2013. *PLoS One* 2015;10(7):e0131353-e53. doi: 10.1371/journal.pone.0131353
43. Statistics South Africa. Mid-year population estimates 2019. Pretoria, South Africa, 2019.
44. Statistics South Africa. Mid-year population estimates 2018. Pretoria, South Africa, 2018.

6 Acknowledgements

The extended *Umoya omuhle* team, institutions, and roles (listed alphabetically by surname):

Name	Institution/s	Role
Siphokazi Adonisi	UCT	Research Assistant
Kathy Baisley	LSHTM; AHRI	Co-investigator
Peter Beckwith	LSHTM; UCT	Research fellow
Fiammetta Bozzani	LSHTM	Co-investigator
Amy Burdzik	UCT	Occupational health
Adrienne Burrough	LSHTM	Project Manager
Nkosingiphile Buthelezi	AHRI	Research Assistant
Xolile Buthelezi	AHRI	Diagnostic Lab Manager
Ruvimbo Chigwanda	UCT	Administration
Christopher Colvin	UCT	Co-investigator
PIP CRAs	AHRI	Clinic research Assistants
Njabulo Dayi	AHRI	Research Data Manager
Arminder Deol	LSHTM	Mathematical modeller
Karina Diaconu	QMU	Co-investigator
Siphephelo Dlamini	AHRI	Nursing Manager
Yutu Dlamini	AHRI	Research Assistant
Raveshni Durgiah	AHRI	Grants office
Anita Edwards	AHRI	Head: Scientific Support
Jennifer Falconer	QMU	Research Assistant
Kitty Flynn	QMU	Administrator
Patrick Gabela	AHRI	Clinical Research Data Coordinator
Dickman Gareta	AHRI	Head: Research Data Management
Awethu Gawulekapa	UCT	Research Assistant
Harriet Gliddon	AHRI; UCL	Research Assistant
Bavashni Govender	UKZN	Administration
Indira Govender	LSHTM; AHRI	Co-investigator
Alison Grant	LSHTM; AHRI	Principal investigator
Meghann Gregg	LSE	Research fellow
Emmerencia Gumede	AHRI	Research Assistant
Sashin Harilall	AHRI	Grants office
Kobus Herbst	AHRI	Chief Information Officer
Tamia Jansen	UCT	Research Assistant
Seonaid Kabiah	UCT	Research Assistant
Idriss Kallon	UCT	Post-doctoral researcher
Aaron Karat	LSHTM	Co-investigator
Hannah Keal	AHRI	Communications
Suzanne Key	UCT	Occupational health
Zama Khanyile	UKZN	Research Assistant
Mandla Khoza	AHRI	Clinic Research Assistant
Nozi Khumalo	AHRI	Systems Engineer

Name	Institution/s	Role
Zilethile Khumalo	AHRI	Research Assistant
Karina Kielmann	QMU	Co-principal investigator
Nondumiso Kumalo	AHRI	Clinic Research Assistant
Richard Lessells	AHRI	Epidemiologist
Nokuthula Lushaba (deceased)	UKZN	Administration
Sithembiso Luthuli	AHRI	Research Assistant
Sinethemba Mabuyakhulu	AHRI	Clinic Research Assistant
Hayley MacGregor	IDS	Co-investigator
Nonhlanhla Madlopha	AHRI	Research Assistant
Aphiwe Makalima	UCT	Administration
Tacha Malaza	AHRI	PIP CRA
Sifundesihle Malembe	AHRI	Research Assistant
Godfrey Manuel	UCT	Transport
Nonhlanhla Maphumulo	UKZN	Administration
Precious Mathenjwa	UCT	Research Assistant
Sanele Mbuyazi	AHRI	PIP CRA
Nicky McCreesh	LSHTM	Co-investigator
Claire McLellan	QMU	Administrator
Simphiwe Mdluli	AHRI	PIP CRA
Thabile Mkhize	AHRI	Transport
Duduzile Mkhwanazi	AHRI	Research Assistant
Zinhle Mkhwanazi	AHRI	Research Assistant
Zodwa Mkhwanazi	AHRI	Research Assistant
Anathi Mngxekeza	UCT	Research Assistant
Tshwaraganang Modise	AHRI	Research Data
Sashen Moodley	AHRI	Microbiology Laboratory Supervisor
Samantha Moyo	UCT	Research Assistant
Silindile Mthembu	AHRI	Clinic Research Assistant
Nozipho Mthethwa	AHRI	Research Assistant
Siphesihle Mthethwa	AHRI	Procurement Coordinator
Sphiwe Mthethwa	AHRI	Research Assistant
Sanele Mthiyane	AHRI	Research Assistant
Vanisha Munsamy	AHRI	Grants office
Sinead Murphy	UCT	Research Assistant
Thomas Murray	AHRI	Research assistant
Senzile Myeni	AHRI	PIP CRA
Tevania Naidoo	AHRI	Procurement
Nompilo Ndlela	AHRI	Research Assistant
Zama Ndlela	AHRI	PIP CRA
Thandekile Nene	AHRI	Research Assistant
Phumla Ngcobo	AHRI	Communications
Nzuzo Ntombela	AHRI	Research Data Systems Service Manager
Sabelo Ntuli	AHRI	GIS Coordinator
Nompumulelo Nyawo	AHRI	Human resources

Name	Institution/s	Role
Phumzile Nywagi	UCT	Research Assistant
Stephen Olivier	AHRI	Statistician
Justin Parkhurst	LSE	Co-investigator
Alex Pym	AHRI	Co-investigator
Yolanda Qeja	UCT	Research Assistant
Anand Ramnanan (deceased)	AHRI	Procurement
Sharmila Rugbeer	UKZN	Administration
Janet Seeley	LSHTM	Co-investigator
Aruna Sevakram	AHRI	Scientific support
Sizwe Sikhakane	AHRI	Transport
Zizile Sikhosana	AHRI	Somkhele Laboratory Supervisor
Theresa Smit	AHRI	Head: Diagnostic Research
Thandeka Smith	UKZN	Research Assistant
Naomi Stewart	LSHTM	Communications
Alison Swartz	UCT	Co-investigator
Amy Thomas	LSHTM	Communications
Siphosethu Titise	UCT	Research Assistant
Anna Vassall	LSHTM	Co-investigator
Marlise Venter	AHRI	Facilities Administrator
Anna Voce	UKZN	Co-investigator
Richard White	LSHTM	Co-investigator
Tom Yates	Imperial	Co-investigator
Precious Zulu	AHRI	Administration
Gimenne Zwama	QMU	Research Fellow

AHRI: Africa Health Research Institute; IDS: Institute of Development Studies; LSE: London School of Economics and Political Science; LSHTM: London School of Hygiene & Tropical Medicine; QMU: Queen Margaret University; UCT: University of Cape Town; UKZN: University of KwaZulu-Natal;
